# Modified Linear Peptides Effectively Silence STAT-3 in Breast Cancer and Ovarian Cancer Cell Lines

**DOI:** 10.3390/pharmaceutics15020666

**Published:** 2023-02-16

**Authors:** Dindyal Mandal, Sandeep Lohan, Muhammad Imran Sajid, Abdulelah Alhazza, Rakesh Kumar Tiwari, Keykavous Parang, Hamidreza Montazeri Aliabadi

**Affiliations:** 1 Center for Targeted Drug Delivery, Department of Biomedical and Pharmaceutical Sciences, Chapman University School of Pharmacy, Harry and Diane Rinker Health Science Campus, Irvine, CA 92618, USA; 2 School of Biotechnology, KIIT Deemed to Be University, Bhubaneswar 751024, India; 3 Faculty of Pharmacy, University of Central Punjab, Lahore 54000, Pakistan; 4 Department of Pharmaceutics, Faculty of Pharmacy, Northern Border University, Rafha 76313, Saudi Arabia

**Keywords:** siRNA, cancer, cholesterol, PEG, peptide, RNA interference, STAT-3, MDA-MB-231, SKOV-3

## Abstract

RNA interference (RNAi) has drawn enormous attention as a powerful tool because of its capability to interfere with mRNA and protein production. However, designing a safe and efficient delivery system in RNAi therapeutics remains challenging. Herein, we have designed and synthesized several linear peptides containing tryptophan (W) and arginine (R) residues separated by the β-alanine (βA) spacer and attached to a lipophilic fatty acyl chain, cholesterol, or PEG. The peptide backbone sequences were: Ac-C-βA-βA-W_4_-βA-βA-R_4_-CO-NH_2_ and Ac-K-βA-βA-W_4_-βA-βA-R_4_-CO-NH_2_, with only a difference in *N*-terminal amino acid. The cysteine side chain in the first sequence was used for the conjugation with PEG2000 and PEG550. Alternatively, the side chain of lysine in the second sequence was used for conjugation with cholesterol or oleic acid. We hypothesized that amphiphilic peptides and optimum fatty acyl chain or PEG could function as an effective siRNA carrier by complementing each structural component’s self-assembly and membrane internalization properties. None of the designed peptides showed cytotoxicity up to 10 µM. Serum stability studies suggested that the newly designed peptides efficiently protected siRNA against early degradation by nucleases. Flow cytometry analysis indicated 50–90% cellular uptake of siRNA using the newly developed modified linear peptides (MLPs). Western blot results revealed more than 90% protein downregulation after targeting STAT3 in MDA-MB-231 and SKOV-3 cell lines. In summary, a new peptide class was developed to safely and efficiently deliver siRNA.

## 1. Introduction

The discovery of RNA inference (RNAi) mechanisms has introduced investigational and therapeutic applications for exogenous short-interfering RNA (siRNA) to temporarily downregulate the expression of the targeted protein [1,2]. Therefore, significant efforts have been made to develop siRNA delivery systems that could bring this approach to clinical use [3,4]. However, there are several challenges impeding the clinical translation of siRNA therapeutics, which include poor serum stability, short half-life (due to rapid degradation and renal elimination), negligible cellular internalization, and endosomal entrapment [5], highlighting the need for a suitable delivery vehicle to facilitate the internalization of siRNA and make it available to the RNAi machinery to elicit target mRNA degradation.

Cell-penetrating peptides (CPPs) provide promising carriers for the intracellular delivery of nucleic acids [6]. CPPs are effective in delivering a wide range of therapeutic molecules, including small molecules, nucleic acids, and proteins with limited toxicity, and are currently further explored in drug delivery studies [6,7,8,9]. A net cationic charge is a common physicochemical property of most CPPs and is known to facilitate membrane internalization [10]. Additionally, the abundance of cationic residues such as arginine (Arg, R) makes CPPs a suitable carrier for anionic cargos such as nucleic acids. A well-defined amphipathic structure is another essential physicochemical property identified for many CPPs [11]. Previous reports have demonstrated the pivotal role of tryptophan (Trp, W) residues in establishing the effective hydrophobic interaction with the lipid bilayer [12].

The potential of various CPPs (TAT, transportan, penetratin, and oligoarginines) to facilitate siRNA internalization was examined via covalent conjugation approaches [13,14]. These strategies have been less successful, most likely due to the resulting chemical modification to siRNA, which could hinder the biological action of the internalized oligonucleotide. Over the past decade, we have reported several Arg-Trp-based cyclic/hybrid peptides having remarkable cell-penetrating properties [8,15,16]. We have thoroughly investigated the effect of the number and relative positions of cationic and hydrophobic residues and noticed that peptides composed of Arg and Trp residues enhanced internalization [15]. After crossing the initial membrane barriers, siRNA complexes need to escape from the endosomal pathway to efficiently function in the cytoplasm. Noticeably, our studies have shown an endocytosis-independent mechanism of uptake for cyclic peptides incorporating tryptophan and arginine residues [15]. In this study, the uptake of fluorescein-labeled [W_5_R_4_K] in leukemia cells (CCRF-CEM) did not significantly change in the presence of endocytosis inhibitors, suggesting a direct translocation mechanism for the uptake.

Various CPPs, including CADY, RICK (Retro-Inverso- CADY-K), TP10, MPG, R9, R9-hLF, PF14, TAT, and LAH4, have been reported for siRNA delivery. Among the CPPs, Retro-Inverso-CADY-K (RICK) and modified TAT peptides were identified to form well-defined self-assembled structures [17,18]. Recently, many reports have demonstrated the self-assembly ability of amphiphilic peptides, which further highlights their suitability for siRNA delivery [19]. Panigrahi et al. observed nanostructure formation with arginine–tryptophan-based cyclic peptide with glycine as a spacer and reported the efficient intracellular delivery of GAPDH siRNA and significant RNAi efficiency [20].

Modified linear peptides (MLPs) represent a class of amphiphilic molecules with an exceptional ability to self-assemble into functional nanostructures, such as micelles and nanovesicles [21]. Therefore, lipidation of amphiphilic peptides is a potential synthetic approach to designing efficient molecular transporters [22,23]. Cholesterol is another well-studied lipid moiety enabling efficient cellular and tissue delivery. The ability of cholesterol to spontaneously intercalate into the lipid membranes upon co-incubation highlights its potential as a critical structural component of the delivery system [24]. Many reports, including one from our group, have demonstrated the key role of cholesterol as a formulation component [16]. Recently, one cholesteryl peptide, Chol-H_3_K_2_, was reported to exhibit a comparable cellular uptake and RNAi effect to PEI or Lipofectamine 2000 without showing any significant cytotoxicity [25]. In another work, Qiu et al. have shown that attachment of PEG with KL4 peptide improves siRNA transfection efficiency in lung epithelial cells [26].

We have also reported siRNA delivery via a wide range of specifically designed peptides, including hybrid cyclic peptides (that incorporate arginine (R) residues in a ring and tryptophan (W) residues in a hydrophobic chain) and cyclic peptides with a disulfide bridge [7,8]. These sequences are designed to resolve the issue of toxicity and poor pharmacokinetic/pharmacodynamic profiles observed with polyethyleneimines (PEI) and poly-L-lysine. However, toxicity could still be a limiting factor in higher concentrations. In this study, building on our previous efforts, where we designed MLPs as a siRNA delivery system, we introduce a novel strategy using peptides containing Trp and Arg residues separated with a β-alanine spacer combined with either lipophilic moieties or PEG chains. The modification of CPPs is required to act as a delivery vector for siRNA to minimize the degradation and mediate the efficient cellular uptake. It is indeed challenging to design efficient short-peptide-based siRNA transporters. We hypothesized that MLPs composed of amphiphilic peptides and optimum fatty acyl chain, or PEG, could function as an effective siRNA carrier by complementing each structural component’s self-assembly and membrane internalization properties. Based on our previous studies, we used four Arg and Trp residues separated by two β-Ala spacers to constitute the peptide region. Oleic acid or cholesterol was used for peptide lipidation.

## 2. Materials and Methods

The resins for solid-phase peptide synthesis and the Fmoc-protected amino acid residues were purchased from AAPPTec (Louisville, KY, USA). Crude peptides were purified using a reverse-phase high-performance liquid chromatography (RP-HPLC) system obtained from Shimadzu (Canby, OR, USA). The preparative C-18 column was used (Waters Bridge, BEH130, 10 µm, 110 Å, 21.2 × 250 mm). Acetonitrile and purified water containing 0.1% TFA (*v*/*v*) were used as solvents. The purification was performed at a flow rate of 8 mL/min, and detection was set at 214 nm. The identity of the molecular structure of peptides was determined by a MALDI-TOF (matrix-assisted laser desorption/ionization-time-of-flight) mass spectrometer (Bruker Inc., Fremont, CA, USA). The matrix for MALDI-TOF mass spectrometry was α-cyano-4-hydroxycinnamic acid.

Fetal bovine serum (FBS), Dulbecco’s Modified Eagle’s Medium (DMEM), and other cell culture supplies were obtained from Life Technologies (Grand Island, NY, USA). Western blot accessories were obtained from Bio-Rad laboratory (Hercules, CA, USA). Re-agents for preparing samples for confocal microscopy were obtained from Vector Laboratories (Burlingame, CA, USA). Monoclonal antibodies to identify GAPDH (Mouse IgG1, Catalogue No. 97166) and STAT3 (Mouse IgG2a, Catalogue No. 9139) were purchased from Cell Signaling Technology, Inc. (Danvers, MA, USA). Secondary antibody (HRP conjugated polyclonal goat IgG detecting Mouse IgGs) was supplied by R&D Systems (Catalogue No. HAF007). siRNA labeled with Alexa fluor 488 (Catalogue No. 1027292), negative control (scrambled) siRNA (Catalogue No. AM4635), and STAT3-targeting siRNA (Catalogue No. SI02662338) were obtained from Life Technologies (Grand Island, NY, USA). Other chemical reagents and solvents were purchased from Millipore-Sigma (Milwaukee, WI, USA) and used without further purification. More details regarding the materials used in the present study have been previously described [7].

### 2.1. Synthesis of the Conjugated Peptides

#### 2.1.1. Synthesis of Modified Linear Peptides (MLPs)

**Figure 1:**
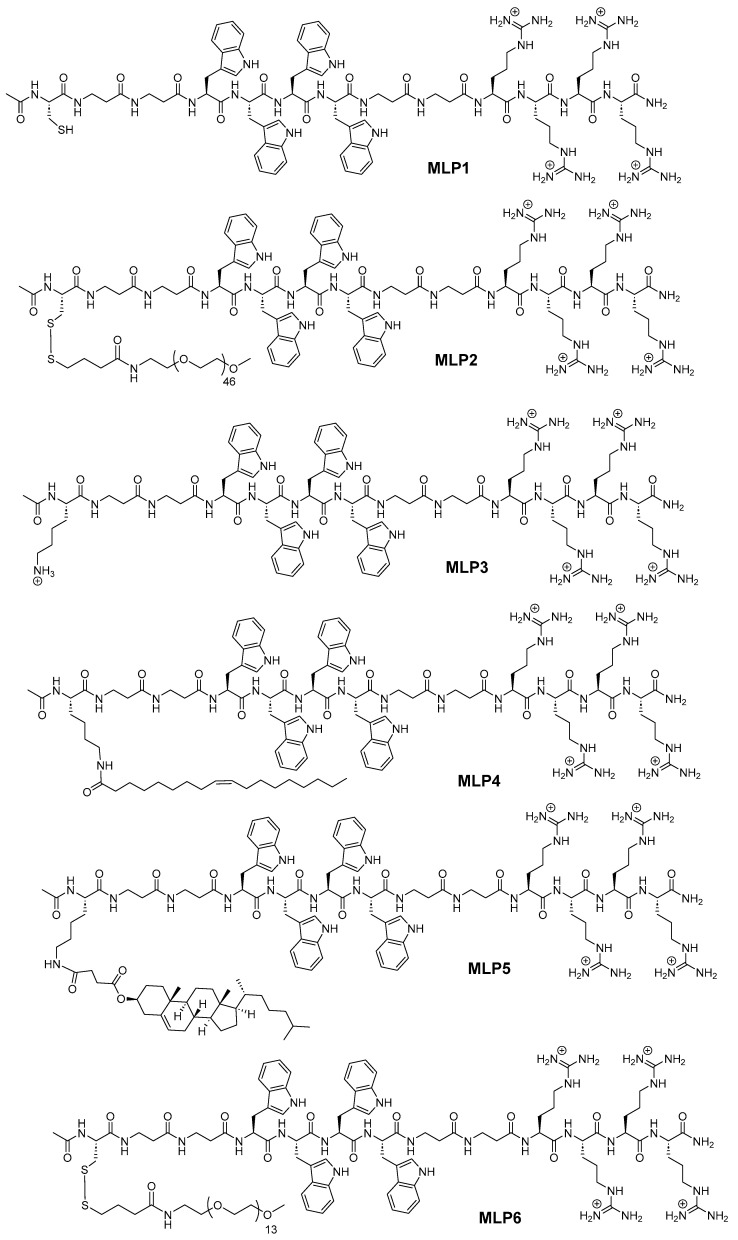
Chemical structures of peptides used in this study.

MLPs were manually synthesized on Rink amide MBHA resin (substitution 0.46 mmol/g) using the standard Fmoc/tBu solid-phase synthesis protocol. The structures of the synthesized peptides are represented in Figure 1, and their sequences are shown in Table 1. Briefly, the resin was allowed to swell in dry DMF for 30 min. Fmoc deprotection was conducted by treating the swelled resin with 20% piperidine in DMF (*v*/*v*) for 20 min. Couplings of natural (Fmoc-L-Arg(Pbf)-OH and Fmoc-L-Trp(Boc)-OH) and unnatural (Fmoc-L-βAla-OH) amino acids were carried out by using Fmoc-L-amino acid (3 equiv.), 1-hydroxy benzotriazole (HOBt, 3 equiv.), and *N*,*N*′-diisopropylcarbiimide (DIC, 3 equiv.), and the reaction mixture was allowed to shake at room temperature for 2–3 h. The coupling of each amino acid was confirmed by a negative Kaiser test. After each coupling, Fmoc-protecting groups were removed by treatment with 20% piperidine in DMF (*v*/*v*). After completing the desired peptide sequence, the *N*-terminus was acetylated by treating the peptidyl resin with a mixture of acetic anhydride pyridine (2:1) in DMF. For side-chain conjugation, Fmoc-L-Lys(Dde)-OH was installed at the *N*-terminal of the peptides. Selective removal of the Dde-protecting group was achieved by treating the peptidyl resin with a solution of hydrazine hydrate in DMF (2%, *v*/*v*). Oleic acid or cholesteryl hemi-succinate were conjugated on the Lys side chain in the presence of HOBt (3 equiv.) and DIC (3 equiv.), and the reaction mixture was allowed to shake at room temperature for 2–3 h. After each coupling and deprotection step, the peptidyl resin was thoroughly washed with DCM and DMF. After completing the desired peptide sequence, the complete cleavage was accomplished in the presence of TFA/water/triisopropyl silane (TIS) (95:2.5:2.5 (*v*/*v*/*v*)), and the crude peptide was precipitated with ice-cooled diethyl ether. After multiple ether washes, the crude peptide was redissolved in acetonitrile/water (1:1 *v*/*v* with 0.5% TFA), and the solution was lyophilized to obtain the crude peptide. The crude peptides were purified using RP-HPLC. The purity of all the peptides was confirmed by analytical HPLC, and mass was analyzed using the MALDI-TOF mass spectrometer. Purity and MALDI mass data of all the peptides are provided in the Supplementary Materials.

#### 2.1.2. Synthesis of PEGylated MLPs

**Scheme 1:**
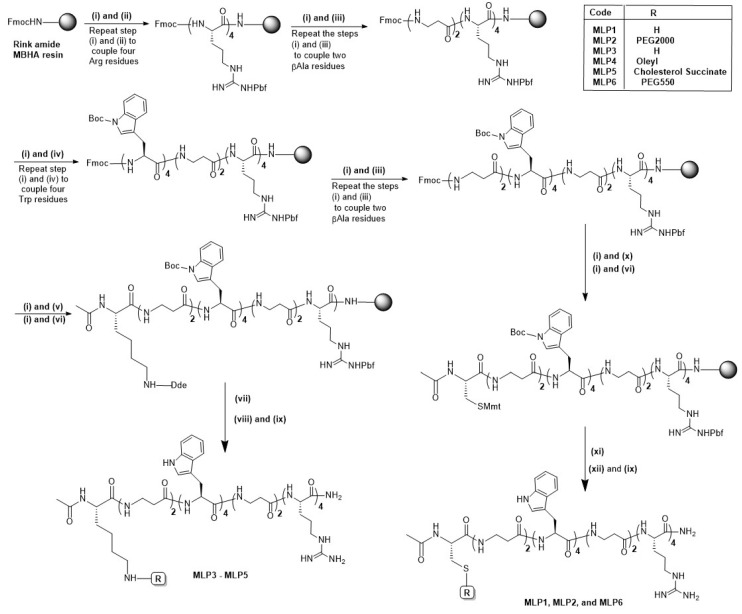
Overview of the steps involved in synthesizing modified linear peptides (MLPs) and pegylated peptides. (i) Fmoc deprotection: 20% piperidine in DMF (2 × 10 min). (ii) Coupling: Fmoc-L-Arg (Pbf)-OH (3 eq.), DIC (3 eq.), HOBt (3 eq.). (iii) Coupling: Fmoc-L-βAla-OH (3 eq.), DIC (3 eq.), HOBt (3 eq.). (iv) Coupling: Fmoc-L-Trp (Boc)-OH (3 eq.), DIC (3 eq.), HOBt (3 eq.). (v) Coupling: Fmoc-L-Lys (Dde)-OH (3 eq.), DIC (3 eq.), HOBt (3 eq.). (vi) Acetylation: acetic anhydride:pyridine:DMF (5:2.5:92.5). (vii) Dde deprotection: 2% hydrazine hydrate in DMF (2 × 5 min.). (viii) Lipidation: Oleic acid (3 eq.) or cholesterol hemi-succinate (3 eq.), DIC (3 eq.), HOBt (3 eq.). (ix) Complete cleavage: TFA:TIPS:H_2_O (95:2.5:2.5; *v*/*v*). (x) Coupling: Fmoc-Cys (Mmt)-OH (3 eq.), DIC (3 eq.), HOBt (3 eq.). (xi) Mmt deprotection: 2% TFA in DCM (2 × 10 min). (xii) PEGylation: activated PEG derivative in DMF (12–14 h).

Synthesis of the pegylated peptides was conducted using solid-phase chemistry, as depicted in the synthesis scheme (Scheme 1). The desired peptide sequence was assembled on Rink amide resin using the reaction condition mentioned in the previous section. Orthogonal-protected Cys residue (Fmoc-Cys(Mmt)-OH) was coupled on the *N*-terminus to PEGylate the peptide by exploiting the side-chain thiol functionality. *N*-terminal acetylation of the resulting peptide sequence was conducted by treating the peptidyl resin with a mixture of acetic anhydride and pyridine (2:1) in DMF. The methoxytrityl group was selectively removed by repeatedly treating the peptidyl resin with 2% TFA in DCM for 10 min. On-resin PEGylation was conducted using preactivated thiol derivatives of PEG (mPEG-3-(2-pyridyldithio) propionic acid, MW 550 and 2000). For the PEGylation reaction, preactivated PEG derivative (2 equiv.) was dissolved in DMF and added to the peptidyl resin. The reaction mixture was kept for shaking for 12–14 h at room temperature. The PEGylated peptides were cleaved from the resin using the cleavage cocktail composed of TFA/water/TIS (95:2.5:2.5 (*v*/*v*/*v*)) and purified by preparative RP-HPLC. The masses of the purified fractions were confirmed by Q-TOF-LC-MS as a multiple-charged peak with a characteristic fragmentation pattern of PEG units (+44 dalton). Table 1 shows the sequences of the synthesized peptides along with the mass data. Mass spectra of all the synthesized peptides (MLP1–MLP6) are provided in the Supplementary Materials.

### 2.2. Binding Affinity

As previously reported, the binding affinity of selected peptides to siRNA was investigated using the SYBR green exclusion assay [16]. The MLPs and scrambled siRNA were mixed in normal saline (in triplicates) with different N/P ratios ranging from 0.05 to 40. The mixture was incubated at room temperature for 30 min to ensure complete complexation of peptides and siRNA. After the incubation time, the complexes were transferred to a black 96-well plate. SYBR green dye solution for this assay was made by diluting 1 part of SYBR green dye with 10,000 parts of purified water. Then, 200 µL of the freshly made SYBR green dye dilution was added to each siRNA complex in the black 96-well plate. The plate was covered with aluminum foil before reading in a micro-plate reader that detected the fluorescent signal (485 nm excitation and 527 nm emission). SYBR green only binds to free siRNA, which significantly enhances the fluorescence signal. By creating a standard curve, the intensity of the fluorescent signal was translated to the concentration of free siRNA, and an indication of the percentage of siRNA bond to the protein. The percentage of the siRNA complexed with the peptide was determined using the following equation:

$$\%\;\mathrm{siRNA}\;\mathrm{bond}\;\mathrm{to}\;\mathrm{peptide}=100-\left(\frac{\mathrm{Fluorescence}\;\mathrm{signal}\;\mathrm{for}\;\mathrm{complexed}\;\mathrm{siRNA}\;}{\mathrm{Fluorescence}\;\mathrm{signal}\;\mathrm{for}\;\mathrm{free}\;\mathrm{siRNA}}\times 100\right)$$

% siRNA bound to the peptide was plotted against a range of nitrogen to phosphate (N/P) ratios used in the experiment, and BR50 (the N/P ratio of the complex required to bind 50% of siRNA) was determined using a sigmoidal model. The negatively charged nucleic acids interact and form complexes with positively charged carriers via interionic interactions. Therefore, the N/P ratio can be a deciding factor, not only in the percentage of siRNA bond to the carrier, but also in how the complexes interact with the cell.

In addition to the SYBR green dye exclusion assay described above, the binding affinity of the peptides to siRNA was also determined using the gel retardation/gel shifting assay. Briefly, the MLPs-siRNA complexes were prepared at different N to P ratios ranging from 0 to 60. The N/P ratio of zero represents free siRNA. The tubes containing complexes were incubated at room temperature for 30 min for complete complex formation. After this incubation time, the complexes were mixed with gel-loading dye. Each of the samples was loaded into the wells of 1% agarose gel with (1 μg/mL) ethidium bromide, and 400 Amperes and 70 Volts were used to run the gel for 20 min. After the run, the gels were visualized under a Bio-Rad imager. The intensity of the bands was quantified by Image Lab software.

### 2.3. Cell Culture

This study used the triple-negative breast cancer cell line MDA-MB-231 (ATCC^®^ HTB-26), and ovarian cancer cells SK-OV-3 (ATCC^®^ HTB 77). The cells were grown in DMEM medium containing 10% *v*/*v* fetal bovine serum and 1% *v*/*v* penicillin (100 U/mL) and streptomycin (100 µg/mL). The incubator for the growth of cells was set at 37 °C with 5% CO_2_ flow. The cells were observed daily under a microscope to determine their growth and health. The experiments were performed when the T75 plate reached 80% confluency.

### 2.4. Cellular Internalization

The cellular uptake of siRNA was determined using Fluorescent-Assisted Cell Sorter (FACS) flow cytometry (BD-FACSVerse; BD Biosciences, San Jose, CA, USA). For this purpose, Alexa fluor 488-labeled siRNA was used in making the peptide-siRNA complexes at different N to P ratios. The cellular uptake experiment was conducted on the breast cancer cell line MDA-MB-231. The detailed protocol is provided in our recently published manuscript [7]. Briefly, cells were seeded in each well of the 24-well plate (approximately 200,000 cells per well) and were allowed to settle in the incubator at 37 °C for 24 h. The peptide and siRNA complexes were made at N to P ratios of 20 and 40, where the final concentration of siRNA in each complex was 36 nM, and were allowed to incubate for 30 min at room temperature. Cells seeded in the 24-well plate were treated with the freshly formed siRNA-peptide complexes and were placed in an incubator for another 24 h to allow the siRNA uptake by the cells. After 24 h of incubation, the media was drained from each well, and the cells were washed three times with HBSS. Following rigorous washing, the cells were trypsinized with colorless trypsin and were quickly visualized under the microscope for their detachment from the surface of the well. The trypsinized cells were fixed in a 3.7% formaldehyde solution, and these suspended cells were investigated via FACS flow cytometry to determine the internalization of Alexa-fluor 488-labeled siRNA. Non-treated cells served as a negative control. The FACS machine was calibrated with a signal gate using non-treated cells, where the fluorescent signal was set at ~1% of the population. The percentage of cells with a fluorescent signal and the mean fluorescence of the cell population were analyzed for each sample.

In order to visualize and locate the internalized siRNA, samples for confocal microscopy were prepared using MDA-MB-231 cells, adopting the protocol described previously by our lab [7]. Briefly, sterile 6-well plates were used, and sterile coverslips were placed at the bottom of each well. MDA-MB-231 cells suspended in 2 mL of media were seeded in each well, and the plates were incubated in an incubator at standard growth conditions. After incubation, the cells were exposed to MLPs-fluorescent-labeled siRNA complexes at a N/P ratio of 40 using the same method described above for the FACS assay. The treated cells were again incubated for 24 h to allow for the internalization of siRNA. After overnight incubation, the media was drained from each well, and the cells were rigorously washed with HBSS three times, followed by fixation with 3.7% formaldehyde in the HBSS solution. Following fixation, the cells were treated with Texas Red for 1 h at room temperature to stain the cell membrane. After 1 h of staining with Texas Red, the cells were stained with DAPI overnight at room temperature in a dark environment to stain the nuclei. Following successful nuclei staining, the coverslips containing fixed stained cells were transferred to slides for viewing under a high-definition confocal microscope (Nikon A1 R).

### 2.5. Serum Stability

The capability of MLPs to protect siRNA against enzymatic degradation in serum was determined using the gel retardation assay described previously [7]. Samples of MLPs-siRNA complexes were made at room temperature with N to P ratios ranging from 0 to 60. The N to P ratio 0 only represents the siRNA. In each of these samples, 25% *v*/*v* fetal bovine serum was added, and the resultant mixture was incubated at 37 °C for 24 h. After incubation, the MLPs-siRNA complexes were dissociated using a heparin competition assay. Heparin was used to dissociate the peptide/siRNA complex as it is anionic in nature and binds with the cationic carrier. The samples were treated with dithiothreitol (DTT, 1 M), followed by treatment with heparin:EDTA (2:3) solution. The concentration of heparin used in this assay was 5% (*w*/*v*), whereas ethylenediaminetetraacetic acid (EDTA) was 0.5 mM. After dissociation of the complexes, the samples were loaded in 1% agarose gel wells containing 1 μg/mL of ethidium bromide. The gel was run for 25 min at 70 V. The gels were finally visualized by ultraviolet illumination using a Bio-Rad imager and the intensity of the bands was quantified using Image Lab software.

### 2.6. Cytotoxicity of Peptide and Peptide/siRNA Complex

The toxicity of the MLPs and MLP-siRNA complexes was determined in MDA-MB-231 cells using the MTS assay, as previously described [7]. The peptides were tested at concentrations ranging from 1 µM to 100 µM, whereas the MLP-siRNA complexes were tested at different N/P ratios ranging from 1 to 80. Briefly, cells were seeded in 96-well plates (approximately 10,000 cells suspended in 100 µL) and were allowed to settle in the incubator for 24 h. After 24 h, the cells were visualized for their adherence and health under a microscope and were exposed to different concentrations of peptides as well as different N to P ratios of MLP-siRNA complexes and were incubated at 37 °C, 5% CO_2_, for 48 h. After incubation, 20 µL of the MTS reagent was added into each well using a multichannel pipette. After 2 h of incubation at 37 °C, the absorbance of each well was determined using the microplate reader (SpectraMax M5, Molecular Devices, San Jose, CA, USA), and the wavelength chosen for absorbance was 490 nm. The cell viability was measured using the following equation:

$$\%\;\mathrm{cell}\;\mathrm{viability}=\frac{\left(\mathrm{OD}\;\mathrm{value}\;\mathrm{of}\;\mathrm{cells}\;\mathrm{treated}\;\mathrm{with}\;\mathrm{peptide}-\mathrm{siRNA}\;\mathrm{complexes}\right)-\left(\mathrm{OD}\;\mathrm{value}\;\mathrm{of}\;\mathrm{culture}\;\mathrm{medium}\right)}{\left(\mathrm{OD}\;\mathrm{value}\;\mathrm{of}\;\mathrm{control}\;\mathrm{cells}\right)-\left(\mathrm{OD}\;\mathrm{value}\;\mathrm{of}\;\mathrm{culture}\;\mathrm{medium}\right)}\times 100$$

### 2.7. Size and ζ-Potential of Peptide/siRNA Complex

MLPs-siRNA complexes were prepared using scrambled siRNA at N/P ratios of 5, 10, 20, and 40, and were analyzed for hydrodynamic diameter and surface charge by a Nano ZS Zetasizer (Malvern, Westborough, MA, USA) at room temperature. The disposable cuvettes were used for the determination of particle size, whereas disposable folded capillary cells were used for zeta potential measurements. All measurements were performed three times using the protocol adopted from our previous study [7]. The ζ-potential was measured at 40 V using the Smoluchowski approximation.

### 2.8. Western Blot

The Western blot assay was used to evaluate the expression of STAT-3 protein in MDA-MB-231 cells using the protocol adopted from our previous study [7]. Briefly, the cells were grown in T25 flasks and treated with MLP-siRNA complexes at N/P ratios of 20 and 40 for 48 h. Following treatment, the cells were harvested as pellets in microtubes and were lysed using RIPA lysis buffer supplanted with a protease inhibitor. The lysate was centrifuged for 15 min at 12,000 rpm at 4 °C to allow the separation of all cell proteins. The supernatant containing all cell proteins was separated in pre-cooled microtubes and the protein was quantified using the standard Bovine Serum Albumin (BSA) assay.

Following the quantification of protein, the samples representing 15 µg of protein were mixed with gel loading dye and loaded in the wells of pre-formed gels and the electrophoresis was carried out at 200 V for 40 min. After the complete run, the gels were transferred to the Western blotting membrane using the Trans-Blot^®^ machine. After successfully transferring gels onto the membrane, the membrane was blocked in 5% Bovine Serum Albumin in TBS-T buffer consisting of 1X tris-buffered saline and 0.1% Tween 20. The membrane blocking was performed for 1 h, followed by washing with TBS-T buffer 3 times each for 5 min. Subsequently, the membrane was incubated with the primary antibody in 1 to 1000 dilution with TBS-T buffer overnight. After overnight treatment with the primary antibody, the membrane was washed with TBS-T buffer four times each for five minutes and was incubated with the secondary antibody (HRP-linked antibody) for one hour. After secondary antibody treatment, the membrane was again washed with TBS-T buffer four times each for 5 min. The same steps were repeated for housekeeping protein (GAPDH). Finally, the membrane was visualized under the Bio-Rad Imager, and the bands were quantified using Image Lab software.

### 2.9. Statistical Analysis

Data obtained from the above-described experiments are shown as the mean ± SD of three independent experiments, unless stated otherwise. Ordinary one-way analyses of variance (ANOVA) with multiple comparisons using Bartlett’s test were conducted to analyze flow cytometry and Western blotting data. Two-way ANOVA was performed using Tukey’s multiple comparisons test to determine the effect of changing the N/P ratio on the particle size and zeta potential. Paired Student’s *t*-test was performed for comparison between the two groups. A *p*-value < 0.05 was considered statistically significant.

## 3. Results and Discussion

The major hurdles in siRNA delivery include stability in the biological environments and efficient delivery inside the cells. In order to circumvent the issues, we have designed a series of linear peptides containing Arg and Trp residues separated with a β-Ala spacer, along with a lipophilic chain, cholesterol, or PEG (Figure 1, Table 1).

### 3.1. Chemistry

We designed a series of modified peptides having a peptide backbone sequentially composed of four consecutive Arg and Trp residues separated by two consecutive β-Ala residues [NH_2_-(βAla)_2_-(W)_4_-(βAla)_2_-(R)_4_-CONH_2_] (Scheme 1). To study the effect of hydrophobic and hydrophilic functionalization on the transfection action of resulting modified peptides, we designed lipidated and pegylated derivatives of the amphiphilic peptide sequence. For lipidation and PEGylation, we installed orthogonally protected Lys (MLP3) and Cys (MLP1) on the *N*-terminus of the primary peptide sequence mentioned above. To determine the effect of aliphatic and aromatic lipidation, we functionalized the Lys side chain via amide linkage with the oleyl chain (MLP4) and cholesterol scaffold (MLP5), respectively. To identify the optimum size of the PEG polymer, we conjugated PEG550 (MLP6) and PEG2000 (MLP2) on the side chain of the Cys via disulfide bridge formation. All lipopeptides and PEGylated peptides were synthesized using a standard solid-phase synthesis protocol. An overview of the steps involved in synthesizing all the peptides is depicted in Scheme 1. Synthesized peptides were characterized and purified using MALDI-TOF mass spectroscopy and RP-HPLC, respectively. Purity was ≥95% confirmed with analytical HPLC.

### 3.2. Biological Assays

#### 3.2.1. Binding Affinity

The binding affinity of the peptides to siRNA was performed using the SYBR green dye exclusion assay to evaluate the interaction between the peptides and siRNA. The results (Table 2) provided the BC_50_ values corresponding to the N/P ratio required for the 50% binding of siRNA. The binding affinity of MLP5 containing cholesterol was found to be the strongest (BC_50_ = 0.905 ± 0.34) among the studied peptides, followed by PEGylated peptides, MLP6 (BC_50_ = 0.937 ± 0.44) and MLP2 (BC_50_ = 0.993 ± 0.28). Oleic acid conjugated peptide (MLP4) also had a comparable binding affinity (BC_50_ = 0.963 ± 0.34). In contrast, MLP1 (BC_50_ = 4.414 ± 0.60) and MLP3 (BC_50_ = 3.752 ± 0.43) have a relatively lower binding affinity with siRNA, which may be due to the lack of structural modifications.

#### 3.2.2. Gel Retardation

**Figure 2:**
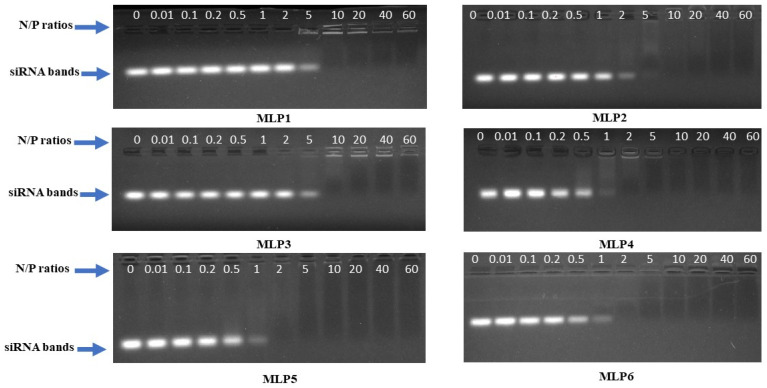
Agarose gel images of the gel retardation assay for the peptides/siRNA complex at different N/P ratios: peptide/scrambled siRNA complexes were formed with different N/P ratios (ranging from 0.01 to 60, in triplicates) to visually confirm the results of the binding affinity experiment using the SYBR green assay.

**Figure 3:**
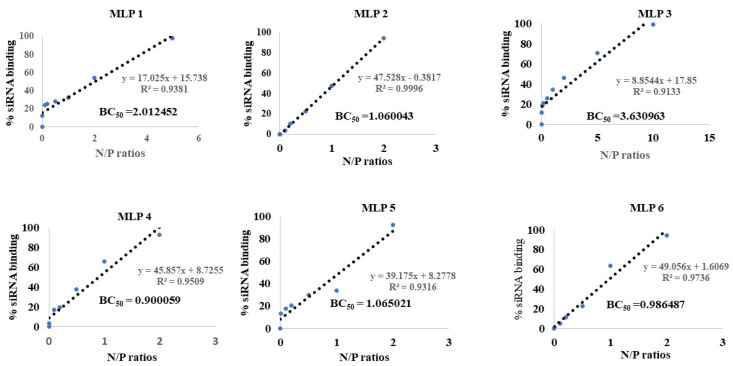
The plots representing the BC_50_ value estimation based on the quantified bands of the gels represented in Figure 2.

To support the data obtained by the SYBR green dye exclusion assay, the binding affinity of the MLPs with siRNA was also determined by the gel retardation assay (Figure 2). For this purpose, the MLPs-siRNA complexes were made in microtubes using purified normal saline at different N to P ratios ranging from 0 to 60. The N to P ratio of 0 represents siRNA only, with no peptide. The solutions in microtubes were allowed to stay at room temperature for 30 min to confer complete complex formation via electrostatic interactions between the MLPs and siRNA. After 30 min of incubation, the gel-loading dye was added into each microtube, and the solution was loaded into the wells of 1% agarose gel containing ethidium bromide. The bands were quantified using Image Lab version 6.0.1 software, and BC_50_ values were determined, as shown in Figure 3. The representative gels are shown in Figure 2. Agarose gel electrophoresis indicated a similar trend, as observed by the SYBR green dye exclusion assay. The results indicated the comparable binding efficiency of MLP2, MLP4, MLP5, and MLP6, which could be due to the presence of conjugating groups such as PEG2000, oleic acid, cholesterol, and PEG550, respectively, present in the peptide sequence. Amphiphilic peptides such as cholesterol peptides are known to significantly condense siRNA compared to their unmodified counterparts [25]. MLP1 and MLP3, which lack conjugation, showed a slightly lower binding affinity and retarded the movement of siRNA. The gel data in Figure 2 represent that all the studied peptides formed adequate complexes with siRNA at an N/P ratio >10.

#### 3.2.3. Particle Size and Zeta Potential

**Figure 4:**
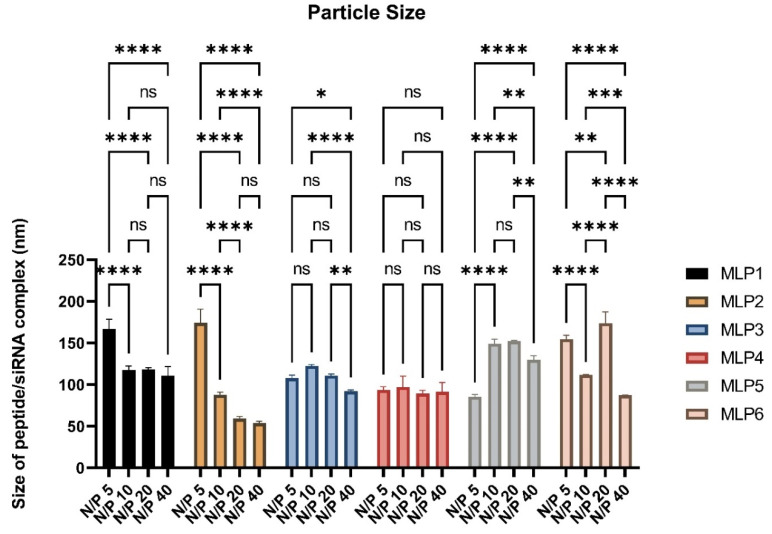
Particle size analysis of the modified linear peptides. Two-way ANOVA was performed using Tukey’s multiple comparisons test to determine the effect of changing the N/P ratio on the particle size. ns = non-significant (*p*-value > 0.05), * *p*-value < 0.05, ** *p*-value < 0.01, *** *p*-value < 0.001, and **** *p*-value < 0.0001. Error bars represent standard deviation (n = 3).

**Figure 5:**
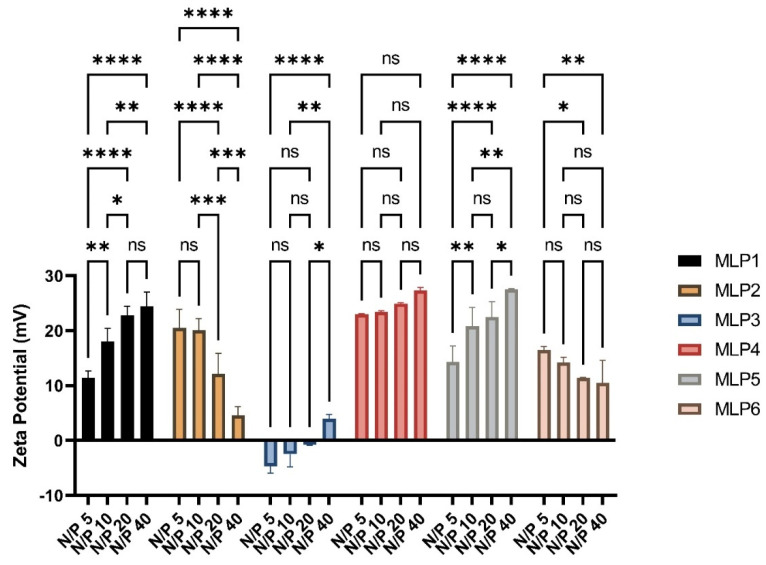
Zeta potential analysis of the modified linear peptides. Two-way ANOVA was performed using Tukey’s multiple comparisons test to determine the effect of changing the N/P ratio on the zeta potential. ns = non-significant (*p*-value > 0.05), * *p*-value < 0.05, ** *p*-value < 0.01, *** *p*-value < 0.001, and **** *p*-value < 0.0001. Error bars represent standard deviation (n = 3).

Characterization of complexes formed by mixing MLPs with siRNA is an important step in understanding how the complexes may interact with biological membranes. Characterizing the complexes at different N to P ratios of 5, 10, 20, and 40 was performed using the Malvern Nano ZS Zetasizer. The hydrodynamic diameter and the ζ-potential of the peptide/siRNA complexes were calculated by the frequency spectrum produced by the Zetasizer and the results are summarized in Figure 4 and Figure 5.

The size of the particle/siRNA complex was found to be <200 nm. This size is considered acceptable for cellular internalization. For MLP1 and MLP2, the size was reduced at higher N/P ratios. The size of complexes formed with MLP3 did not show any significant difference at various N/P ratios. The size of complexes formed with MLP5 increased at higher N/P ratios of 10 and 20, while for MLP6, the highest size was observed at the N/P ratio of 20, and the size decreased at a N/P ratio of 40. Overall (including all N/P ratios for each peptide), a significant difference in the size of particles formed with different peptides included in this study was not observed (one-way ANOVA). However, it was observed that MLP1, MLP2, and MLP6 showed a different trend (decrease in size with an increase in the N/P ratio). These specific structures belong to a different class of peptides that contain thiol or disulfide groups. It is possible that the free thiol group in MLP1s forms a disulfide crosslink, which is known to provide stability to complex structures [27]. We believe that upon interactions of the peptides with siRNA, the peptides lose their linearity because of the interactions of positively charged residues with the negatively charged siRNA. Transmission electron microscopy (TEM) and/or scanning electron microscope (SEM) studies could be beneficial in further exploring this observation and are planned for the continuation of this study.

The ζ-potential represents the composition of the complexes and could affect the stability of the colloidal system. The ζ-potential of the MLP1/siRNA complexes increased with an increase in N/P ratios from 5 to 40 (ζ-potential from 11.4 to 24.4 mV), which was expected due to an increase in the proportion of the positively charged component of the complexes. A similar trend was also observed for complexes formed with MLP3, MLP4, and MLP5; however, the increase was not significant for MLP4, and the ζ-potential never reached the positive values observed for other complexes (−4.7 mV to +3.95 mV for complexes formed with MLP3 and different N/P ratios; Figure 5). Interestingly, the surface charge of complexes formed with PEGylated peptides decreased with increasing the N/P ratios. This result indicates a shielding effect for the long chain of hydrophilic PEG. These data are consistent with the decreased size at higher N/P ratios for MLP2 and MLP6. Overall (including all N/P ratios for each peptide), the ζ-potential of complexes formed with MLP3 was significantly lower compared to all other peptides (one-way ANOVA), which was unexpected and requires more investigation.

#### 3.2.4. Serum Stability

A key challenge in translating siRNA therapeutics from bench to bedside is their rapid destruction by endogenous nucleases, which shortens their blood circulation time to a few minutes and hence limits their therapeutic use [28]. Hence, a delivery system that could enhance siRNA’s half-life and protect it from the nucleases is a much-sought endeavor. We investigated the MLPs for their ability to protect siRNA when exposed to harsher conditions, such as by exposure to 25% *v*/*v* fetal bovine serum for 24 h. After exposure to FBS overnight, the heparin competition assay was used, which is an anionic component that dissociates siRNA from the cationic delivery systems [29].

**Figure 6:**
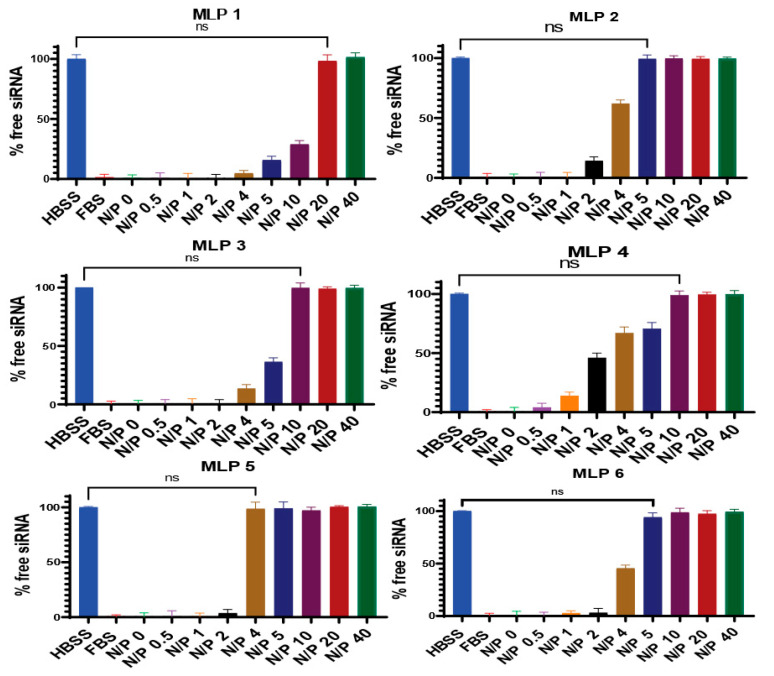

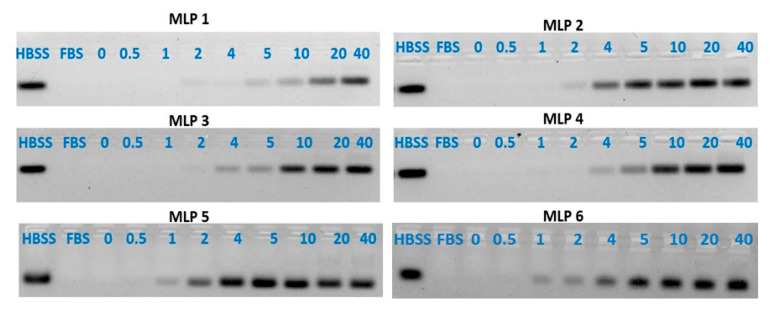
Serum stability of siRNA after complex formation with peptides. Heparin sulfate dissociated siRNA from the peptide/siRNA complexes (FBS 25%, incubation time 24 h). The band representing the negative control (scrambled siRNA exposed to saline for 24 h at 37 °C) was quantified as 100%. Data are presented as the average of triplicate samples, and the error bars represent standard deviation. ns = non-significant. The bands were quantified using Image Lab software version 6.0.1.

Based on the zeta potential results, it was decided to perform serum stability experiments using MLP1, MLP2, MLP3, MLP4, MLP5, and MLP6. The results are depicted in Figure 6. All studied peptides showed comparable efficiency toward the protection of siRNA, while MLP1, an unmodified peptide, was less efficient at all N/P ratios. MLP5 provided comparable protection to the negative control at a N/P ratio of 5. Additionally, the PEGylated MLP2 showed significant protection at a N/P ratio of 5, whereas MLP3, MLP4, and MLP5 provided efficient protection to siRNA at N/P ratios of 10 and above.

#### 3.2.5. Cellular Internalization Using FACS

Cellular internalization of siRNA is one of the necessary steps in RNA interference and developing efficient siRNA delivery systems. Upon the intracellular delivery of siRNA, endosomal escape and release of siRNA from the delivery vehicle are the other necessary steps toward successful post-transcriptional mRNA targeting and degradation.

**Figure 7:**
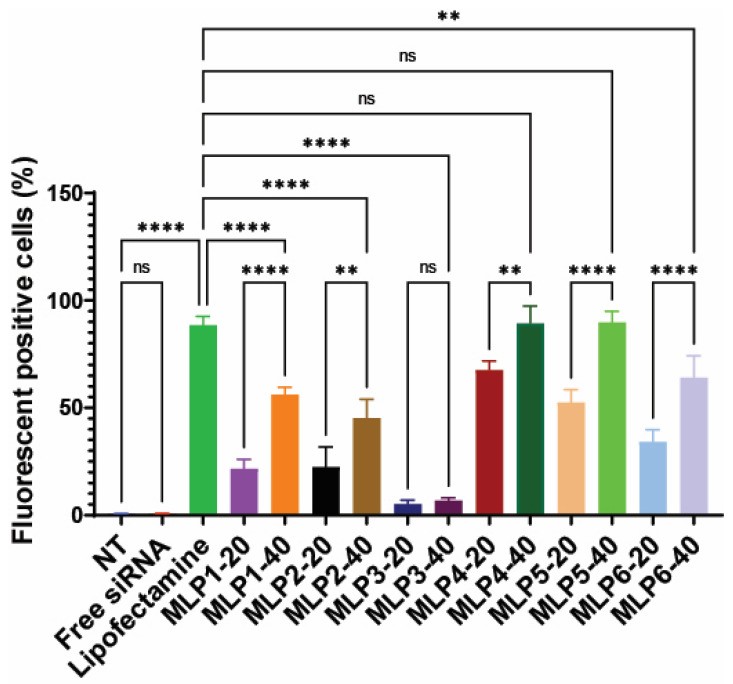
Cellular uptake of Alexa Fluor 488-labeled siRNA in MDA-MB-231 cells in the presence of peptides (MLP1–MLP6) and Lipofectamine. The number adjacent to the peptide code indicates the N/P ratio. The concentrations of siRNA and Lipofectamine for all the groups were 36 nM and 1 µg/mL, respectively (incubation time: 24 h). Bars represent the mean value (n = 3), and the error bars represent standard deviations. ns = non-significant (*p*-value > 0.05), ** *p*-value < 0.001, and **** *p*-value < 0.0001.

siRNA internalization was determined in the triple-negative breast cancer cell line MDA-MB-231, as it is reported to be a rather difficult cell line to transfect [30]. We selected this cell line to determine the uptake of Alexa Fluor 488-labeled siRNA using MLP-siRNA complexes at N/P ratios of 20 and 40. These ratios were chosen for each peptide based on the results of gel retardation and serum stability assays (Figure 7). Additionally, we compared the transfection efficiency of the peptides with the commercial reagent Lipofectamine 2000.

All the tested peptides except MLP3 showed significant cellular internalization of siRNA. As anticipated, unmodified MLP3 did not show any cellular internalization. As reflected in the zeta potential measurement, MLP3 lacks a sufficient positive charge to interact with the cell membrane. As described above for MLP3, the ζ-potential of the complex changed from −4.7 mV to +3.95 mV at N/P ratios from 5 to 40, suggesting a weak surface charge on the complex. In contrast, another unmodified peptide, MLP1, shows ~50% siRNA uptake, possibly due to the cysteine residue that generates a disulfide bond and promotes cellular internalization. Other modified peptides bearing high zeta potential can easily interact with the membrane. Among the peptides screened, MLP4, MLP5, and MLP6 showed more than 50% siRNA uptake, whereas MLP1 and MLP2 exhibited uptake in the range of 30% to 45%. The ζ-potential of MLP1/siRNA enhanced with the increase in N/P ratios from 5 to 40 (ζ-potential from 11.4 to 24.4 mV), and the presence of positive surface charges explains the differential behavior of MLP1 and MLP3 in cellular internalization.

#### 3.2.6. Confocal Microscopy

**Figure 8:**
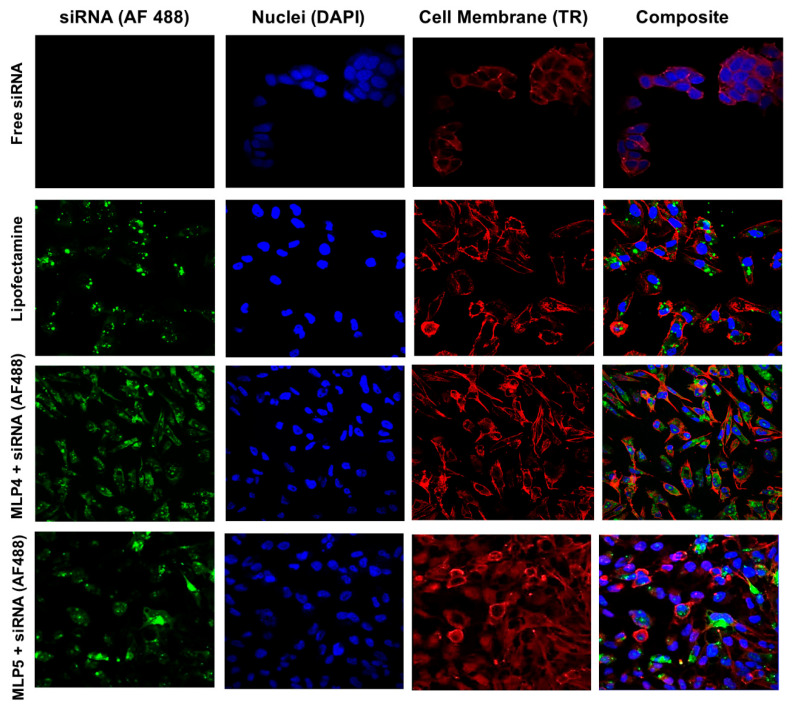
Representative confocal images of the MDA-MB-231 cells following treatment with Alexa Fluor 488-labeled siRNA-peptide (MLP4 or MLP5) complexes. Free siRNA-treated cells were used as a negative control, while Lipofectamine-siRNA complex served as a positive control.

To visualize the cellular internalization of siRNA, confocal microscopy was used to track the siRNA signal (Figure 8). The cell membrane and nuclei were stained with sulforhodamine101 (Texas Red) and DAPI, respectively. Free siRNA and Lipofectamine 2000 were used as negative and positive controls, respectively. Bright green fluorescence emerged from the intracellular region, indicating significant internalization of siRNA in the cytosol in the presence of the MLP4 and MLP5, which was comparable to the commercial reagent Lipofectamine 2000.

#### 3.2.7. Cytotoxicity

**Figure 9:**
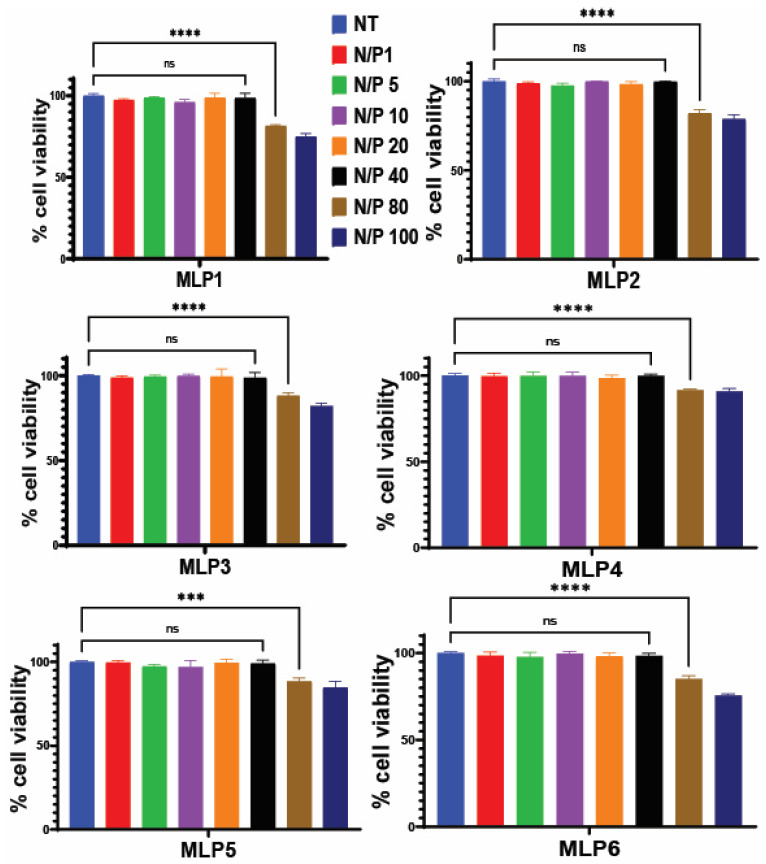
Cytotoxicity of peptide-siRNA complexes in MDA-MB-231 cells at different N/P ratios (incubation time: 48 h). Graphs represent mean normalized values (based on No Treatment, or the NT group, n = 3). Error bars indicate standard deviation. ns = non-significant (*p*-value > 0.05), *** *p*-value < 0.0005 and **** *p*-value < 0.0001.

One of the criteria for developing a delivery system is the nontoxicity of the carrier. To assess the cytotoxicity of the peptides, different concentrations of the peptides were tested in MDA-MB-231 cells. NT stands for non-treated cells that served as a negative control. DMSO (35% *v*/*v*) was used as a positive control (data not represented in the graphs). No significant effect on the cell viability was observed for all the studied peptides until a 10 µM concentration, as shown in Supplementary Figure S2. However, there was a significant reduction in cell viability observed above a 10 µM concentration, which was why the maximum N/P ratio of 40 (Figure 9) was chosen for this study, where the maximum concentration of any of the peptides was 8.64 µM. Overall, a few discrepancies were observed in comparing the toxic effects of free peptides and peptide/siRNA complexes. Electrical charge is one of the deciding factors for the interaction with the cell membrane, which can be one of the causes for toxicity as well (due to a compromise in the integrity of the membrane). Therefore, this discrepancy can be explained by the difference in the way that peptides and peptide/siRNA complexes interact with the cells.

#### 3.2.8. Targeting a Specific Protein

Although multiple signaling factors are involved in the proliferation and metastasis of cancer cells, signal transducer and activator of transcription 3 (STAT3) has been implicated as a key factor in the propagation of several cancer cell types [31]. Based on the importance of STAT3, this protein was chosen as a model protein for the RNAi study.

Various delivery systems for targeting STAT3 have been developed to date. However, the peptide-based strategy remains unexplored [32]. Thus, we have used the newly designed peptides for STAT3 delivery.

**Figure 10:**
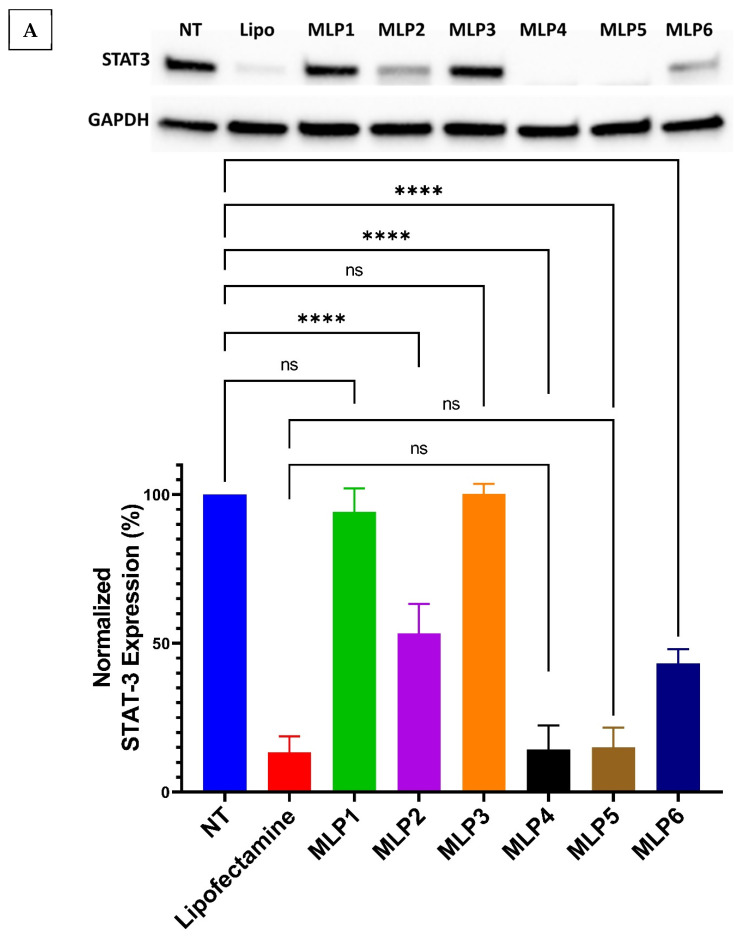

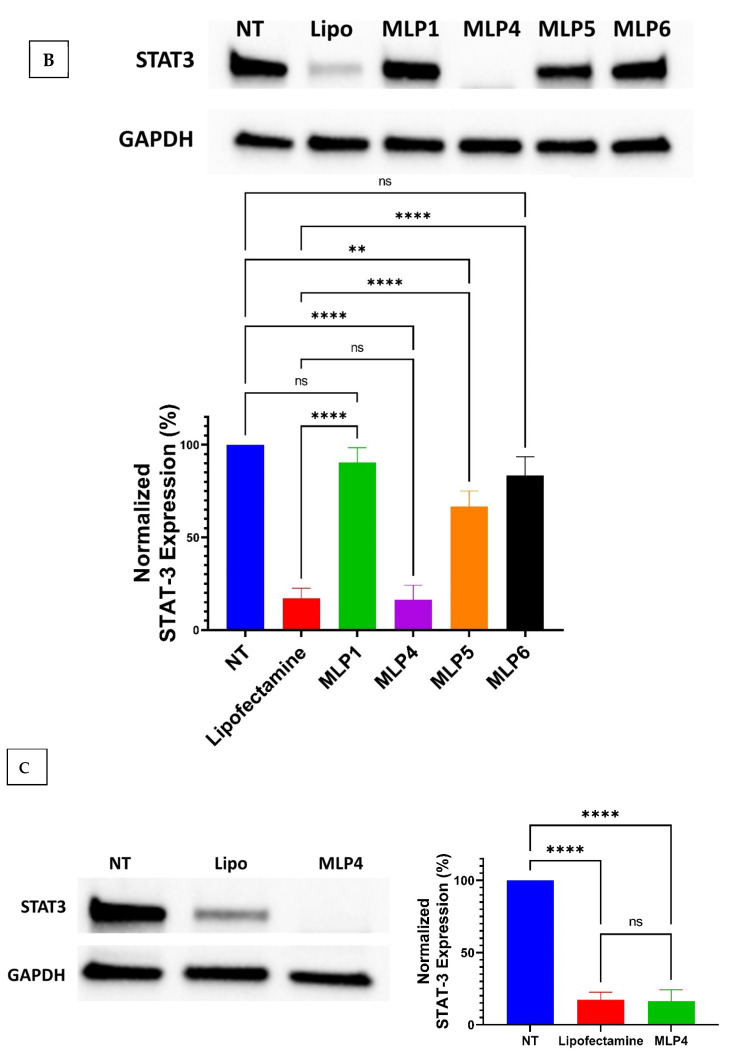
Comparison of STAT 3 protein downregulation efficiencies of lipopeptides at N/P ratios of 40 (**A**) and 20 (**B**) in MDA-MB-231 cells and the downregulation efficiency of MLP4 at a N/P ratio of 20 in SK-OV3 cells (**C**). Error bars indicate standard deviation (n = 3). ns = non-significant, ** *p*-value < 0.001, and **** *p*-value < 0.0001.

Western blot analysis was performed to monitor the protein expression level to confirm the effect of peptide/siRNA complex in MDA-MB-231 cells. Among the screened peptides, MLP4 and MLP5 showed >90% downregulation of STAT3 in MDA-MB-231 cells at a N/P ratio of 40, comparable to the commercially available transfection agent, Lipofectamine (Figure 10A). In contrast, unmodified peptides such as MLP1 and MLP3 did not exhibit any protein downregulation, suggesting the crucial role of lipophilic groups such as oleic acid and cholesterol in siRNA delivery and the release of functional siRNA in the cytosol. Peptides conjugated with a PEG moiety (MLP2 and MLP6) also showed significant downregulation of STAT3. However, they were found to be less efficient than MLP4 and MLP5. This could be attributed to the lower availability of free siRNA because of the presence of a long PEGylation chain in the peptide sequence.

Furthermore, to check the efficiency of the peptides at a lower concentration, cells were treated with modified peptides (MLP2, MLP4, MLP5, MLP6) at lower N/P ratios (20). Interestingly, MLP4 was found to be efficient for >90% STAT3 downregulation in the same cell line at a lower N/P ratio (Figure 10B), while other peptides, including MLP5, were not as efficient compared to N/P 40. The N/P ratio is an important factor in the efficiency of siRNA carriers and could potentially create significant differences. The N/P ratio can affect the stability of siRNA in the serum (before uptake into the cells), the toxicity of the nanoparticles, and perhaps most importantly, the release of siRNA molecules in the cytoplasm. This could potentially explain the discrepancies observed here.

To confirm the efficiency of RNAi in a different cell line, MLP4 was used for targeting STAT3 in the ovarian cancer SK-OV3 cell line. The result indicates that more than 90% downregulation of STAT3 occurs in SK-OV3 (Figure 10C), suggesting the broad applicability of the peptide for protein downregulation. Figure 10C depicts the expression of STAT3 upon the delivery of the STAT3 siRNA/MLP4 complexes, which shows approximately 85% downregulation of the protein.

## 4. Conclusions

We have screened a new class of modified linear peptides consisting of Trp and Arg residues separated by β-Ala and fatty acyl chain or PEG chain for siRNA delivery. While our experimental design is associated with specific limitations (e.g., using MTS to examine cytotoxicity and lack of a positive control in this experiment, or a lack of more extensive experiments to identify the exact mechanism of cellular internalization), the presented study revealed some interesting findings about the relationship between the structural design and the characteristics of the carriers. Among the peptides, modified sequences with fatty acyl groups effectively delivered siRNA to breast cancer cells and efficiently silenced the functional protein. The results indicate the formation of a substantial complex between the peptides and siRNA, which efficiently penetrated the cell membrane and reached the cytosol. Functional protein downregulation in breast cancer cells was highly efficient with minimal cytotoxicity, and RNAi efficiency was comparable to a commercially available transfecting reagent. Validation of the newly generated delivery system in another cell line, such as ovarian cancer cells, eliminates the possibility of cell-specific siRNA delivery. These newly synthesized MLPs will be a significant impetus in discovering peptide-based carriers possessing excellent nucleic acid transfection capabilities with added serum stability and potential for in vivo applications.

## Figures and Tables

**Table 1:** Synthesized MLPs and MALDI-TOF/ESI-TOF characterization.

Sr. No.	Conjugate	Sequence	Chemical Formula	Exact Mass	Found (M/Z)
1	MLP1	AcNH-C-βA-βA-W_4_-βA-βA-R_4_-CONH_2_	C_85_H_118_N_30_O_14_	1814.9164	1815.4735 [M]^+^
2	MLP2	AcNH-C(PEG2000)-βA_-_ βA-W_4_-βA_-_ βA-R_4_-CONH_2_	C_184_H_315_N_31_O_62_S_2_	4015.1890	[M+44+H]^3+^ [M+44+H]^4+^ [M+44+H]^5+^ [M+44+H]^6+^
3	MLP3	AcNH-K-βA_-_ βA-W_4_-βA_-_ βA-R_4_-CONH_2_	C_94_H_137_N_27_O_11_	1840.0022	1841.4814 [M+H]^+^
4	MLP4	AcNH-K(Oleyl)-βA-βA-W_4_-βA_-_ βA-R_4_-CONH_2_	C_106_H_157_N_31_O_15_	2104.2475	2104.5391 [M+H]^+^
5	MLP5	AcNH-K(Cholesterol)-βA_-_ βA-W_4_-βA_-_ βA-R_4_-CONH_2_	C_119_H_173_N_31_O_17_	2308.3626	2309.1060 [M+H]^+^
6	MLP6	AcNH-C(PEG550)-βA_-_ βA-W_4_-βA_-_ βA-R_4_-CONH_2_	C_118_H_183_N_31_O_29_S_2_	2562.3239	850.4340 [M+44+H]^3+^

**Table 2:** BC_50_ values of synthesized peptides.

Peptide Code	Peptide Sequence	BC_50_ ± SD
MLP1	AcNH-C-βA-βA-W_4_-βA-βA-R_4_-CONH_2_	4.414 ± 0.60
MLP2	AcNH-C(PEG2000)-βA-βA-W_4_-βA-βA-R_4_-CONH_2_	0.993 ± 0.28
MLP3	AcNH-K-βA-βA-W_4_-βA-βA-R_4_-CONH_2_	3.752 ± 0.43
MLP4	AcNH-K(oleyl)-βA-βA-W_4_-βA-βA-R_4_-CONH_2_	0.963 ± 0.34
MLP5	AcNH-K(cholesterol)-βA-βA-W_4_-βA-βA-R_4_-CONH_2_	0.905 ± 0.23
MLP6	AcNH-C(PEG550)-βA-βA-W_4_-βA-βA-R_4_-CONH_2_	0.937 ± 0.44

## Data Availability

The data presented in this study are available on request from the corresponding author. The data belong to AJK Biopharmaceuticals.

## Supplementary Materials

The following supporting information can be downloaded at: https://www.mdpi.com/article/10.3390/pharmaceutics15020666/s1: Supplementary Figure S1 (MALDI-TOF mass spectrometry spectra), Supplementary Figure S2 (analytical HPLC chromatograms), Supplementary Figure S3 (cytotoxicity of the peptides as free molecules), and Supplementary Figure S4 (the unedited Western Blot images).
