# Correction: Mandal et al. Modified Linear Peptides Effectively Silence STAT-3 in Breast Cancer and Ovarian Cancer Cell Lines. *Pharmaceutics* 2023, *15*, 666

**DOI:** 10.3390/pharmaceutics17101324

**Published:** 2025-10-13

**Authors:** Dindyal Mandal, Sandeep Lohan, Muhammad Imran Sajid, Abdulelah Alhazza, Rakesh Kumar Tiwari, Keykavous Parang, Hamidreza Montazeri Aliabadi

**Affiliations:** 1 Center for Targeted Drug Delivery, Department of Biomedical and Pharmaceutical Sciences, Chapman University School of Pharmacy, Harry and Diane Rinker Health Science Campus, Irvine, CA 92618, USA; 2 School of Biotechnology, KIIT Deemed to Be University, Bhubaneswar 751024, India; 3 Faculty of Pharmacy, University of Central Punjab, Lahore 54000, Pakistan; 4 Department of Pharmaceutics, Faculty of Pharmacy, Northern Border University, Rafha 76313, Saudi Arabia

In the original publication [1], there was a mistake in Figures 6 and 8 as published. The authors would like to make the following updates to the published article [1] to ensure clarity and completeness of the data presentation. During a post-publication review of the article, it was discovered that Figures 6 and 8 contained mistakes due to an honest oversight. Specifically, we noted that the serum stability data for MLP4 and MLP6 in Figure 6 were inadvertently duplicated. Furthermore, the confocal microscopy images for Free siRNA and MLP5 + siRNA in Figure 8 were mistakenly assembled [1] using our previously published images [2]. These duplications occurred during the preparation of the final Figures. We have now corrected Figures 6 and 8 with the correct representative images, which accurately reflect the experimental results. We confirm that these corrections reflect the true experimental results.

Importantly, the conclusions of the study remain unchanged and valid. The corrections to Figures 6 and 8 do not affect any other data or the interpretation of the results in the paper. The authors emphasize our commitment to scientific accuracy and integrity, and we sincerely apologize for any confusion or inconvenience caused by these errors. We have worked diligently to ensure that the scientific record is promptly corrected.

The authors confirm that aside from the corrected Figure 6 and Figure 8, no other parts of the manuscript are affected. We remain committed to upholding the highest standards of scientific rigor and thank the readers for their understanding. All authors have read and agreed to this correction.

The authors state that the scientific conclusions are unaffected. This correction was approved by the Academic Editor. The original publication has also been updated.

## Figures and Tables

**Figure 6:**
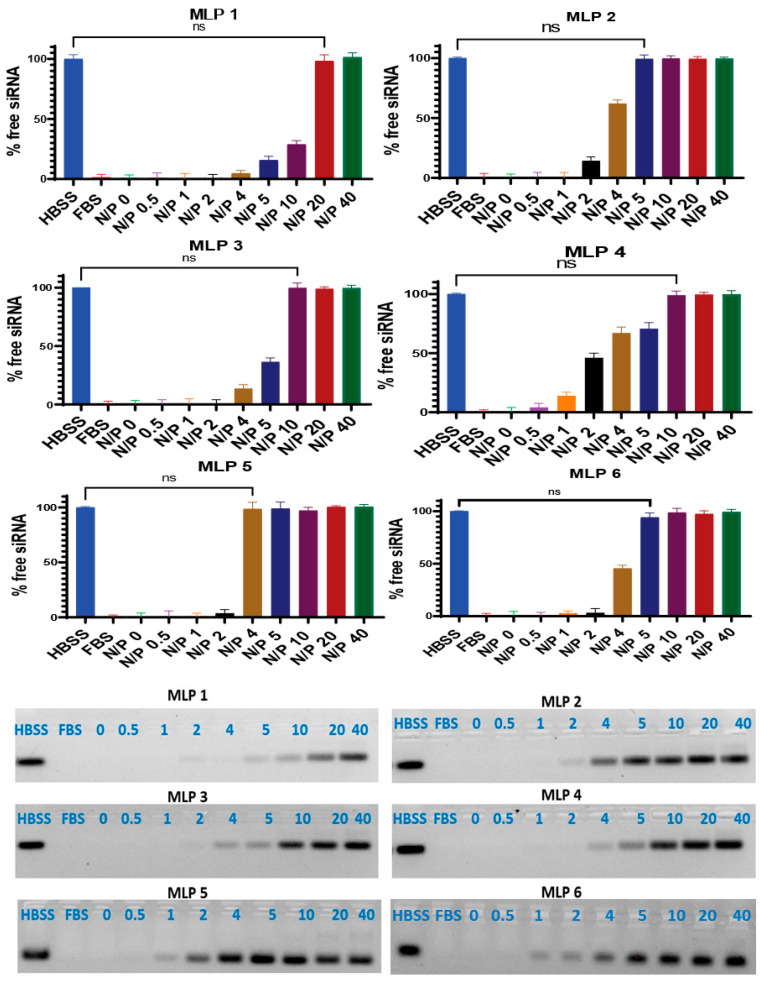
Serum stability of siRNA after complex formation with peptides. Heparin sulfate dissociated siRNA from the peptide/siRNA complexes (FBS 25%, incubation time 24 h). The band representing the negative control (scrambled siRNA exposed to saline for 24 h at 37 °C) was quantified as 100%. Data are presented as the average of triplicate samples, and the error bars represent standard deviation. ns = non-significant. The bands were quantified using Image Lab software version 6.0.1.

**Figure 8:**
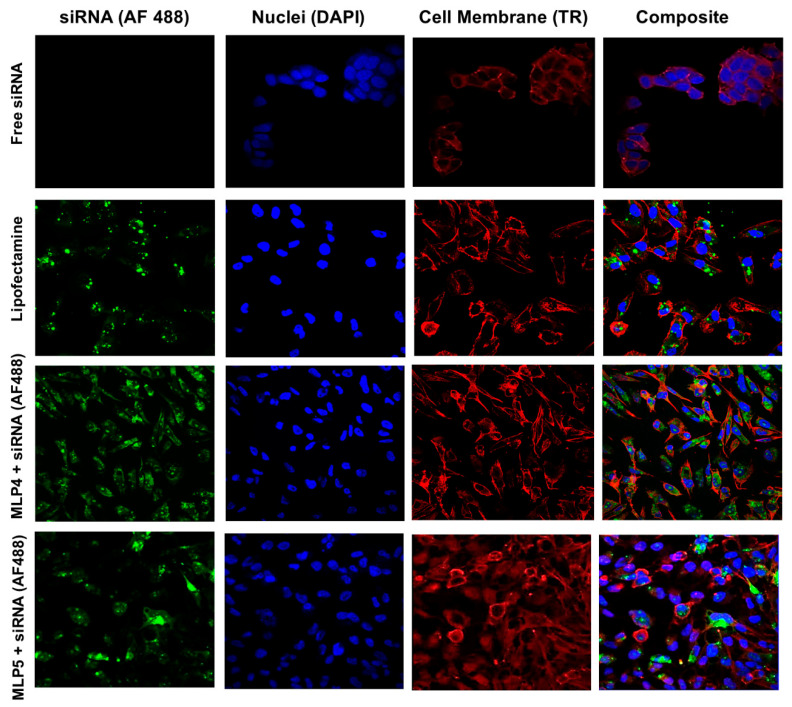
Representative confocal images of the MDA-MB-231 cells following treatment with Alexa Fluor 488-labeled siRNA-peptide (MLP4 or MLP5) complexes. Free siRNA-treated cells were used as a negative control, while Lipofectamine-siRNA complex served as a positive control.
