# Supplementary material for: Modified Linear Peptides Effectively Silence STAT-3 in Breast Cancer and Ovarian Cancer Cell Lines
Source: Pharmaceutics. 2023 Feb 16;15(2):666. doi: 10.3390/pharmaceutics15020666 (PMC9962452; doi:10.3390/pharmaceutics15020666)
Supplement: Supplementary file 1 [file pharmaceutics-15-00666-s001.zip › pharmaceutics-2149977-supplementary.pdf]

Supporting Information

Modified Linear Peptides Effectively Silence STAT-3 in Breast Cancer and Ovarian Cancer Cell lines

Dindyal Mandal [1,2], Sandeep Lohan [1], Muhammad Imran Sajid [1,3], Abdulelah Alhazza [1,4], Rakesh Kumar Tiwari [1,*], Keykavous Parang [1,*] and Hamidreza Montazeri Aliabadi [1,*]

1 Center for Targeted Drug Delivery, Department of Biomedical and Pharmaceutical Sciences, Chapman University School of Pharmacy, Harry and Diane Rinker Health Science Campus, Irvine, CA 92618, USA; dmandal@kiitbiotech.ac.in (D.M.); lohan@chapman.edu (S.L.); msajid@chapman.edu (M.I.S.); alhazza@chapman.edu (A.A.)

2 School of Biotechnology, KIIT Deemed to Be University, Bhubaneswar 751024, India

3 Faculty of Pharmacy, University of Central Punjab, Lahore 54000, Pakistan

4 Department of Pharmaceutics, Faculty of Pharmacy, Northern Border University, Rafha 76313, Saudi Arabia

* Correspondence: tiwari@chapman.edu (R.K.T.); parang@chapman.edu (K.P.); montazer@chapman.edu (H.M.A.); Tel.: +1-(714)-516-5483 (R.K.T.); +1-714-516-5489 (K.P.); +1-714-516-5492 (H.M.A.); Fax: +1-(714)-516-5481 (R.K.T.) & (K.P.) & (H.M.A.)

Supplementary Figure S1. Mass Spectra of Synthesized Peptides.

A. MLP1

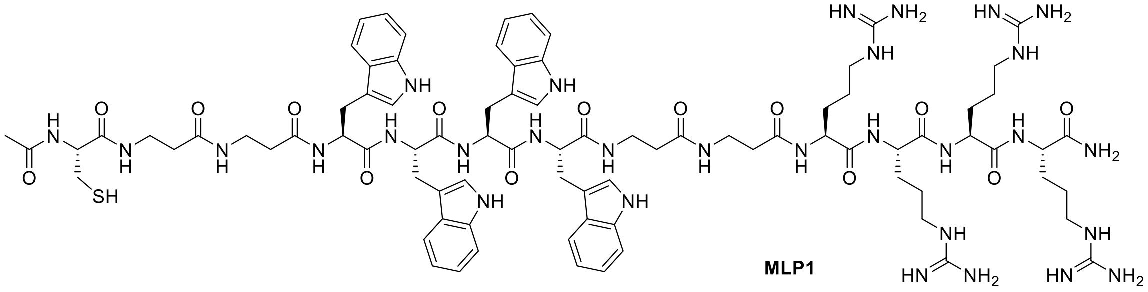

Chemical Formula: $C_{85}H_{118}N_{30}O_{14}S$
Exact Mass: 1814.9164

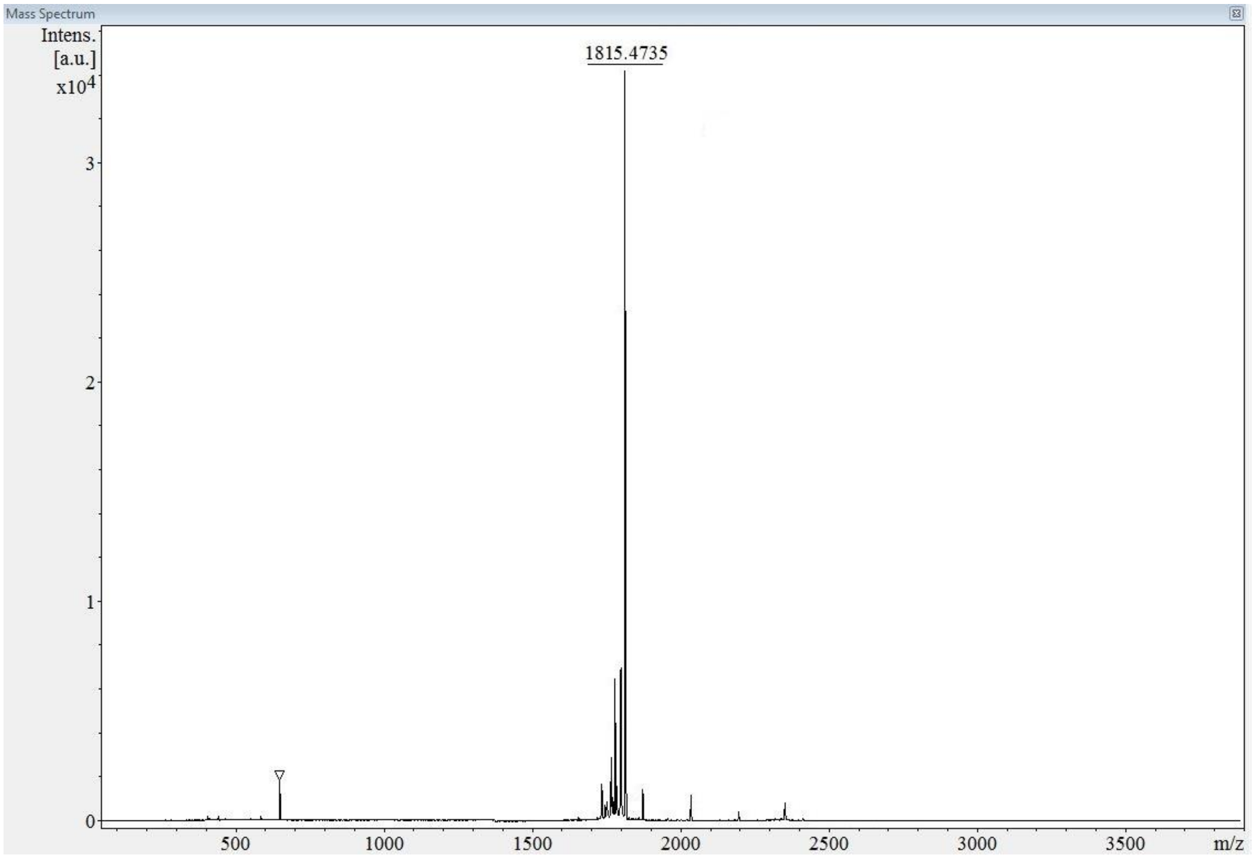

B. MLP2

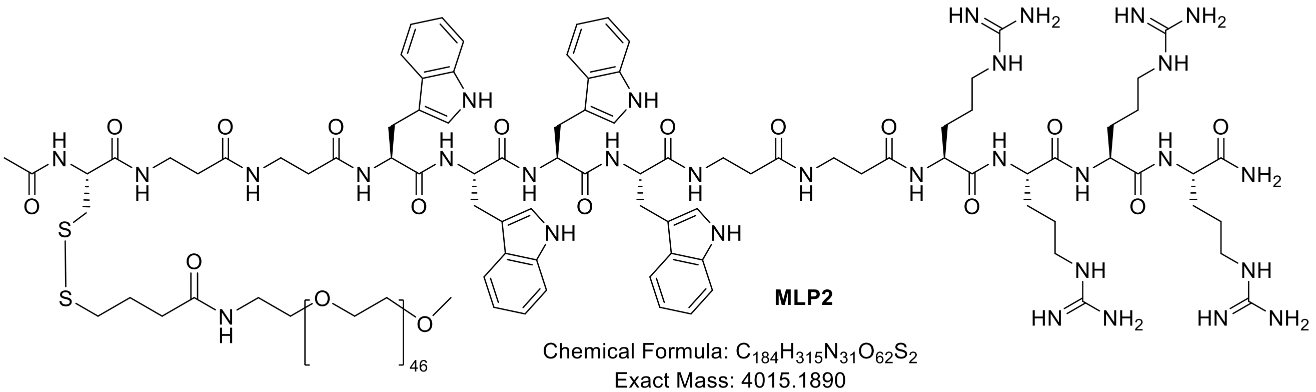

Chemical Formula: $C_{184}H_{315}N_{31}O_{62}S_2$
Exact Mass: 4015.1890

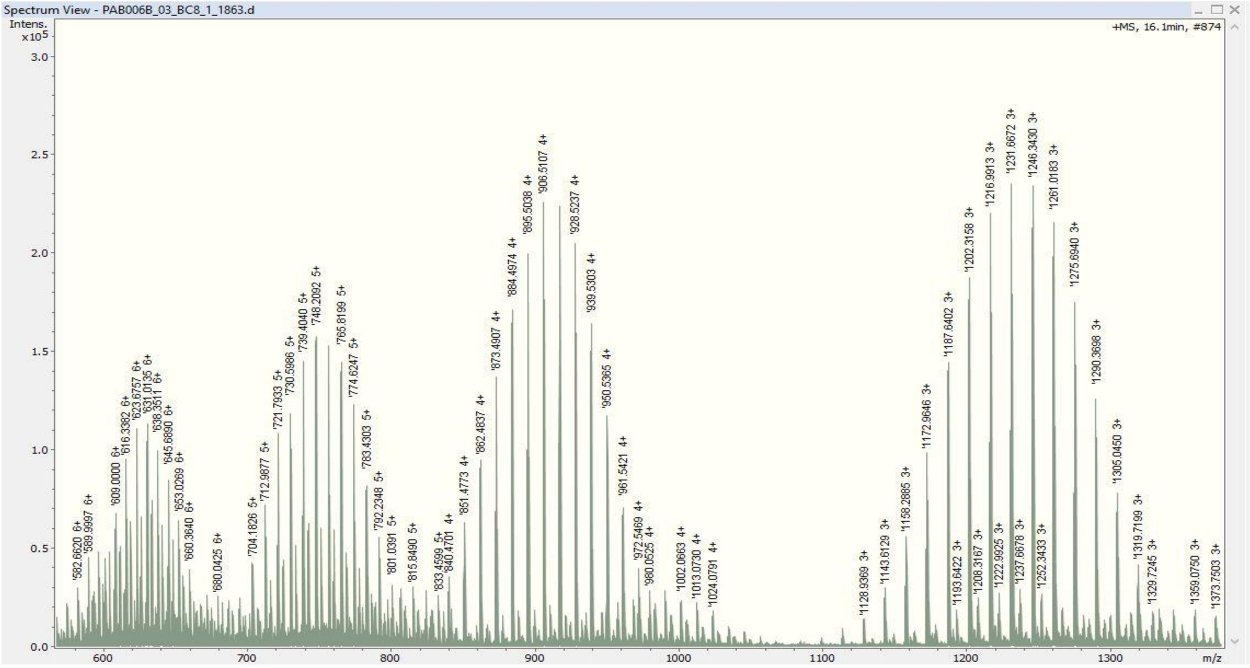

C. MLP 3

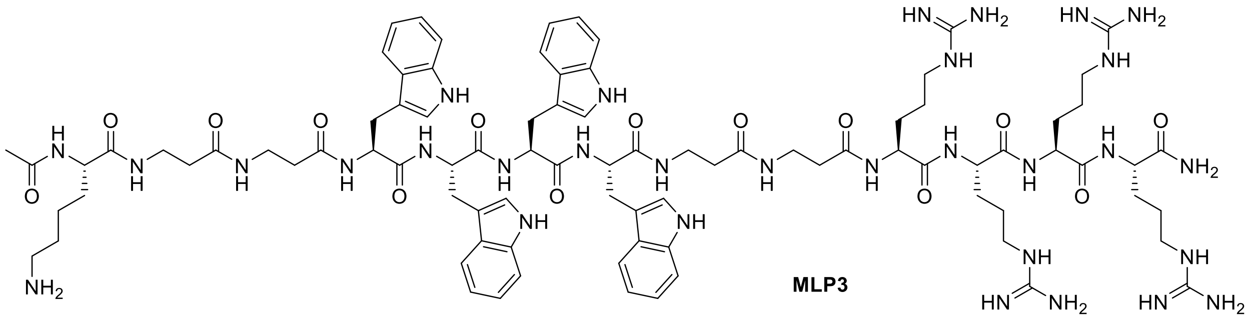

Chemical Formula: $C_{88}H_{125}N_{31}O_{14}$
Exact Mass: 1840.0022

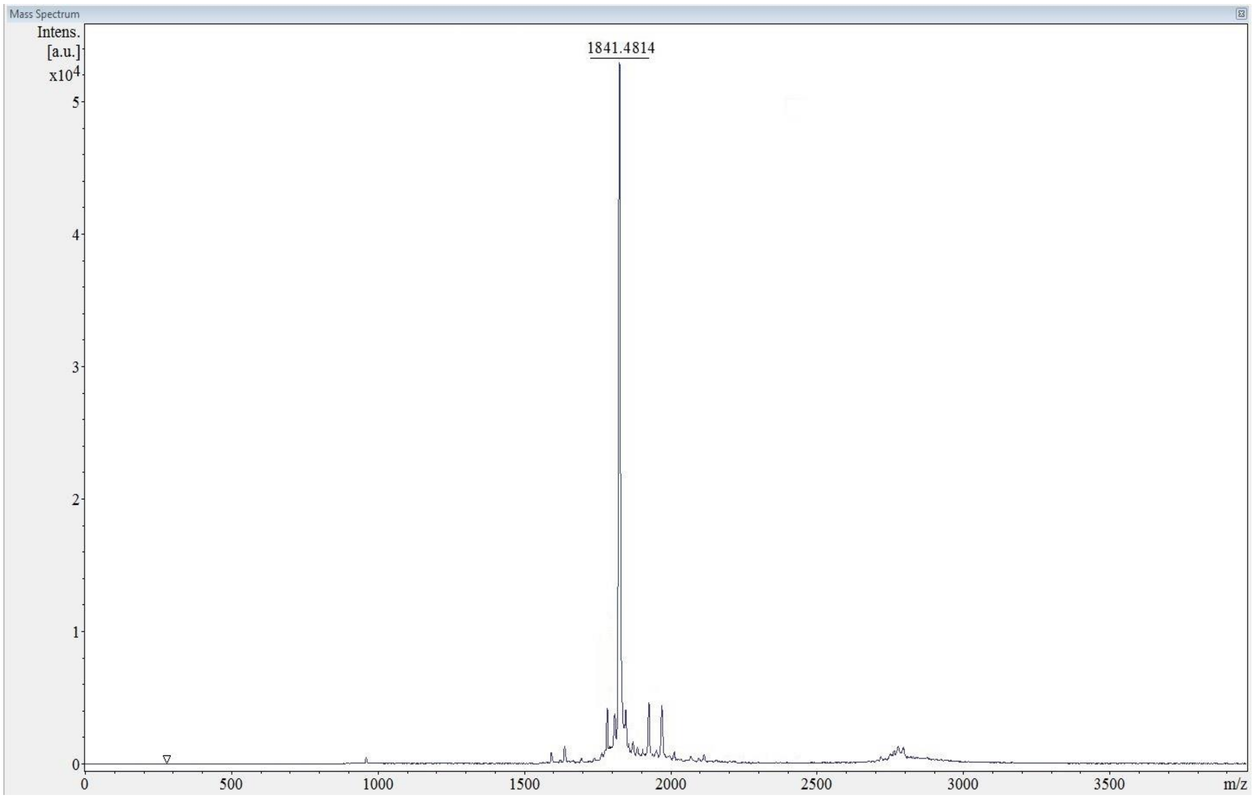

D. MLP4

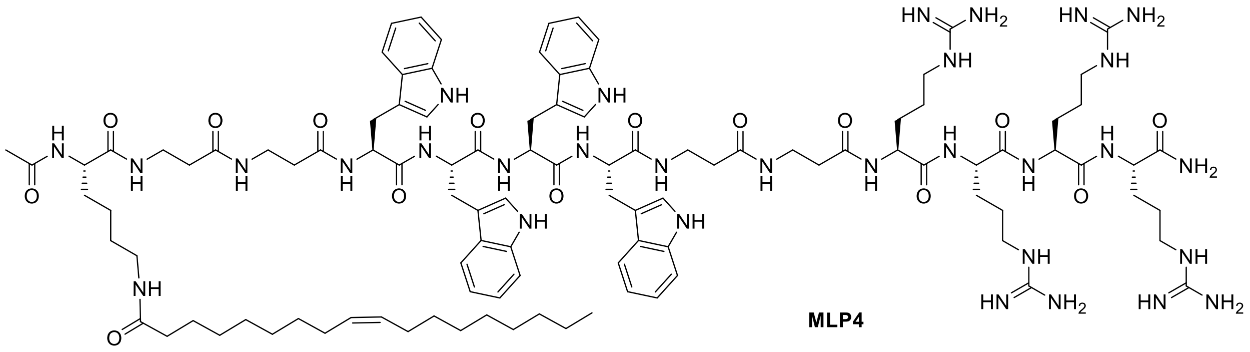

Chemical Formula: $C_{106}H_{157}N_{31}O_{15}$
Exact Mass: 2104.2475

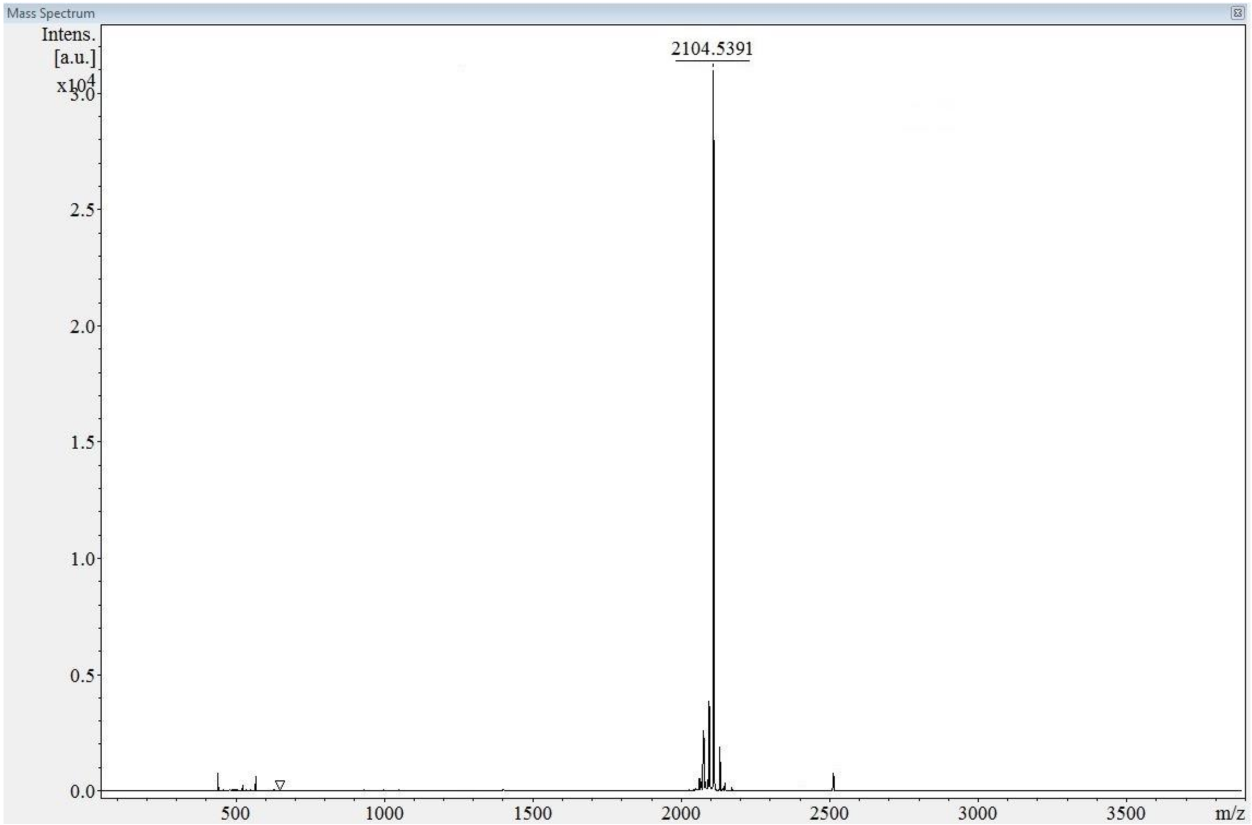

E. MLP5

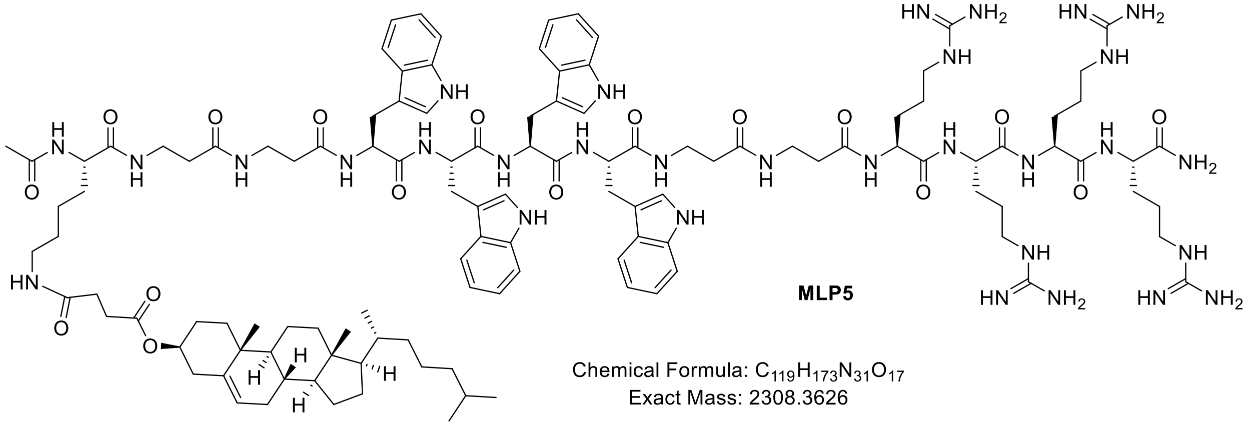
MLP5
Chemical Formula: $C_{119}H_{173}N_{31}O_{17}$
Exact Mass: 2308.3626

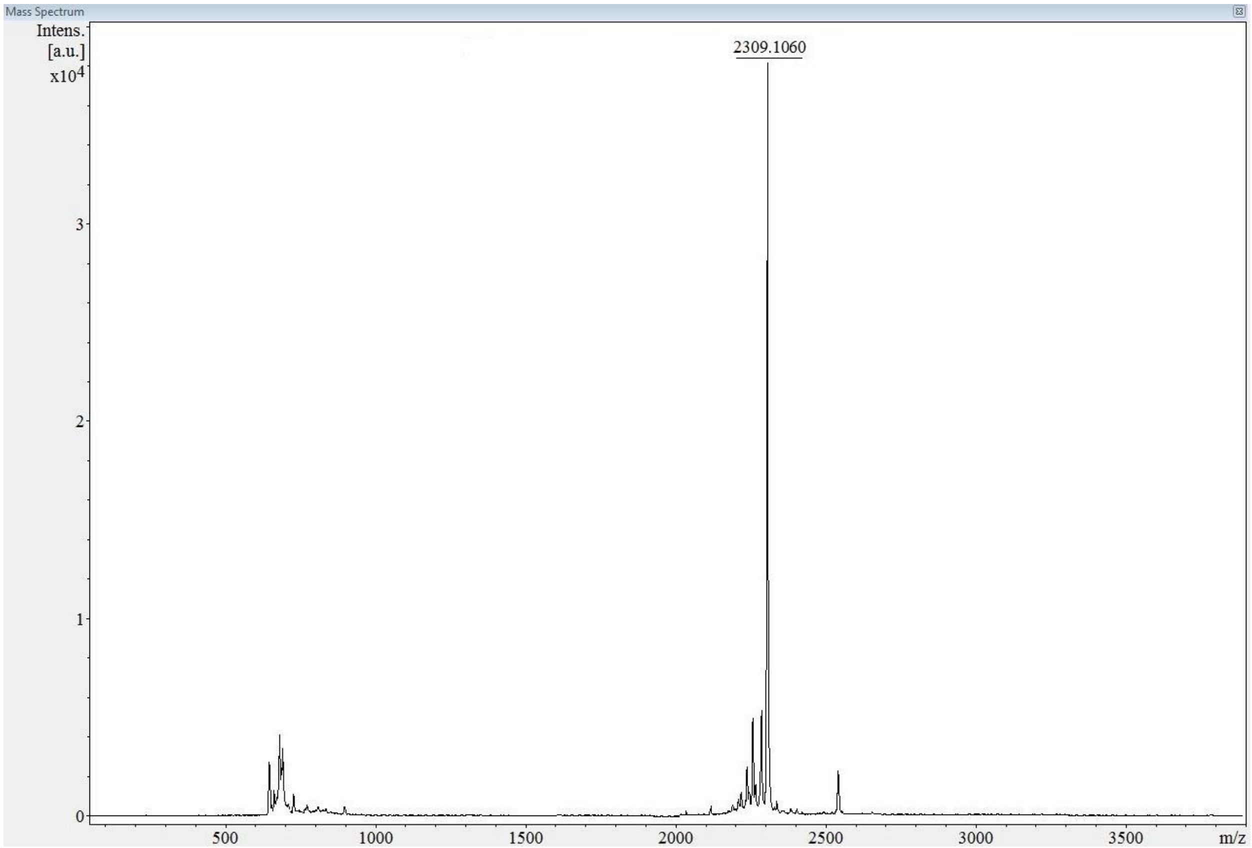
Mass Spectrum
Intens.
[a.u.]
x10^4
2309.1060
3
2
1
0
500
1000
1500
2000
2500
3000
3500
m/z

F. MLP6

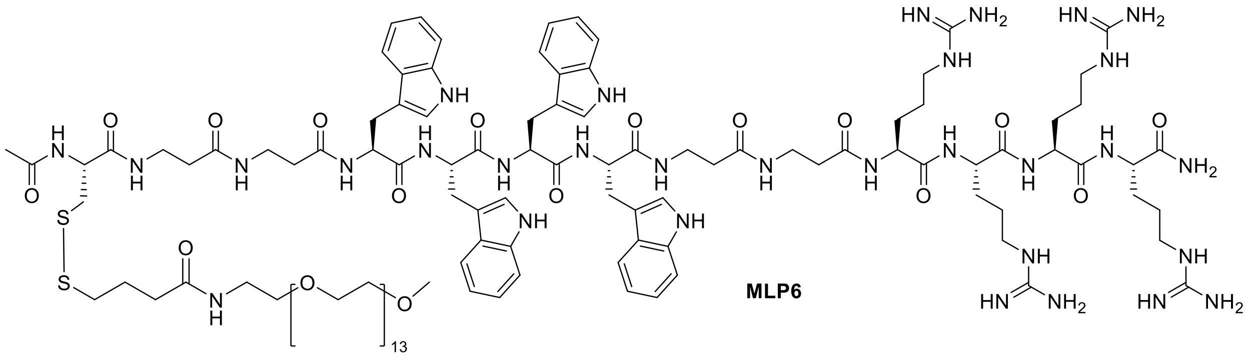

Chemical Formula: $C_{118}H_{183}N_{31}O_{29}S_2$
Exact Mass: 2562.3239

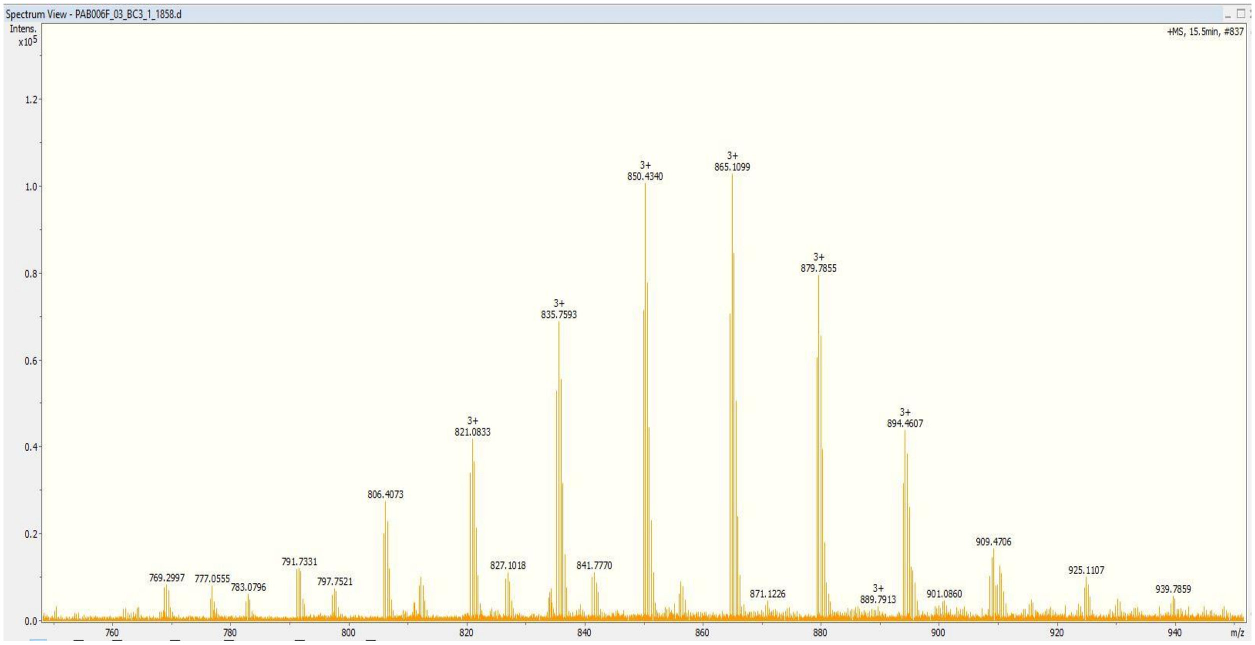

Supplementary Figure S2. HPLC data for non-PEGylated Synthesized Peptides.

MLP-1

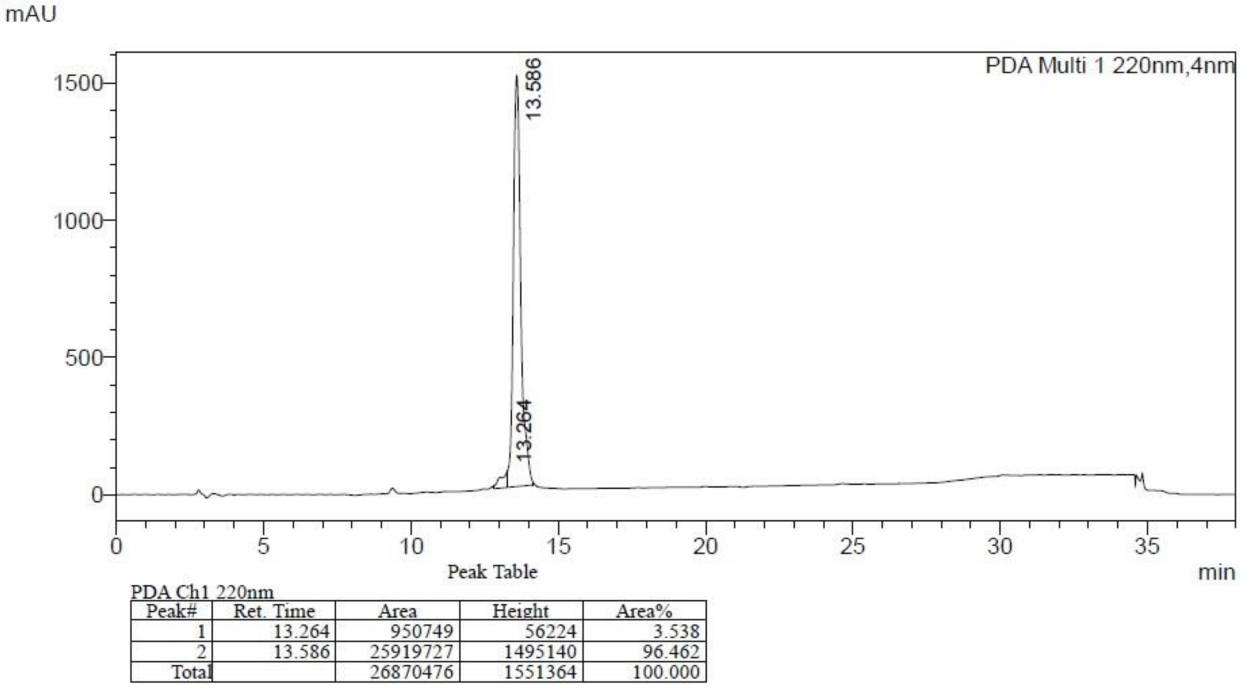

Peak Table

PDA Ch1 220nm

Peak#	Ret. Time	Area	Height	Area%
1	13.264	950749	56224	3.538
2	13.586	25919727	1495140	96.462
Total		26870476	1551364	100.000

MLP-3

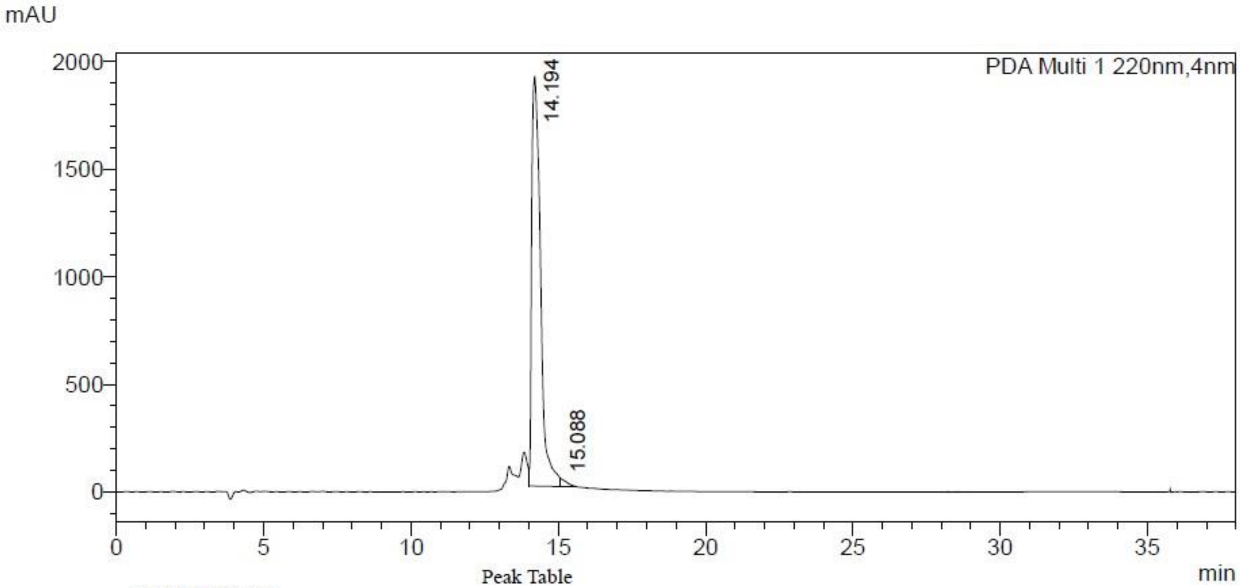

Peak Table

PDA Ch1 220nm

Peak#	Ret. Time	Area	Height	Area%
1	14.194	38872947	1901150	98.697
2	15.088	513293	37430	1.303
Total		39386240	1938580	100.000

MLP-4

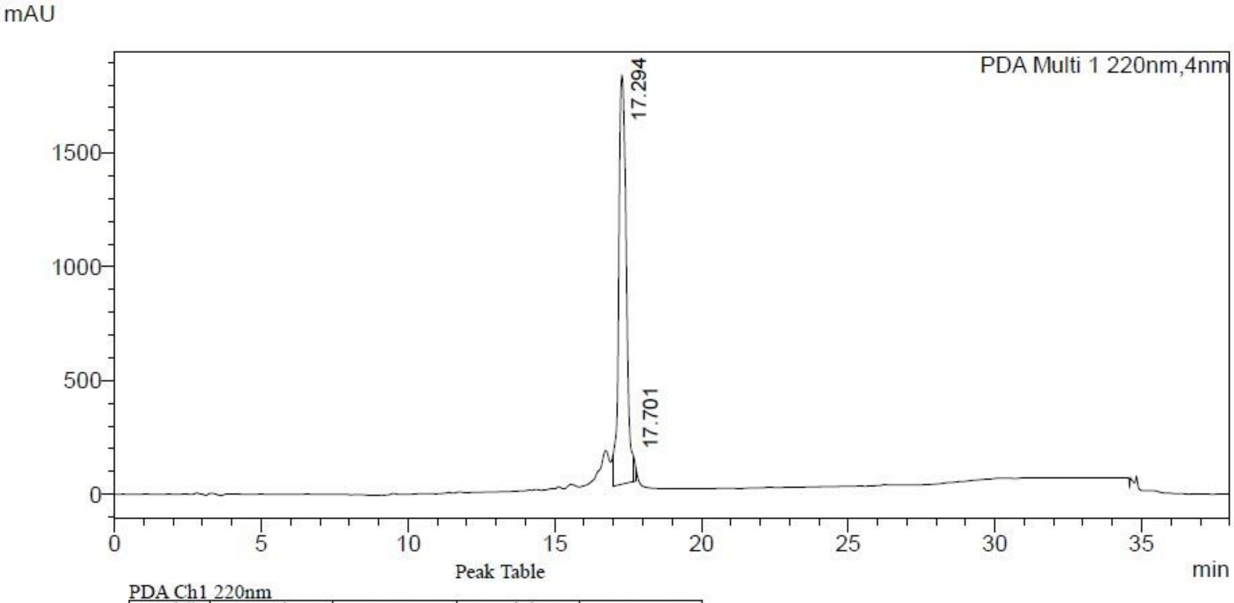

Peak Table

PDA Ch1 220nm

Peak#	Ret. Time	Area	Height	Area%
1	17.294	31754214	1796445	98.822
2	17.701	378541	106565	1.178
Total		32132755	1903010	100.000

MLP-5

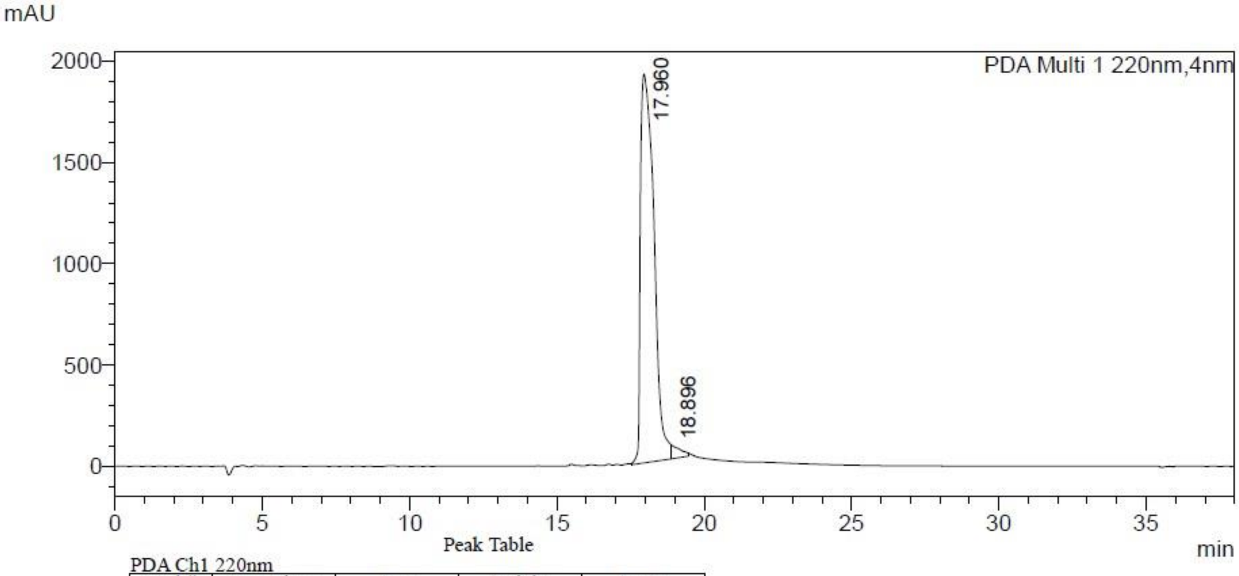

Peak Table

PDA Ch1 220nm

Peak#	Ret. Time	Area	Height	Area%
1	17.960	60590278	1916862	97.781
2	18.896	1375284	66713	2.219
Total		61965562	1983576	100.000

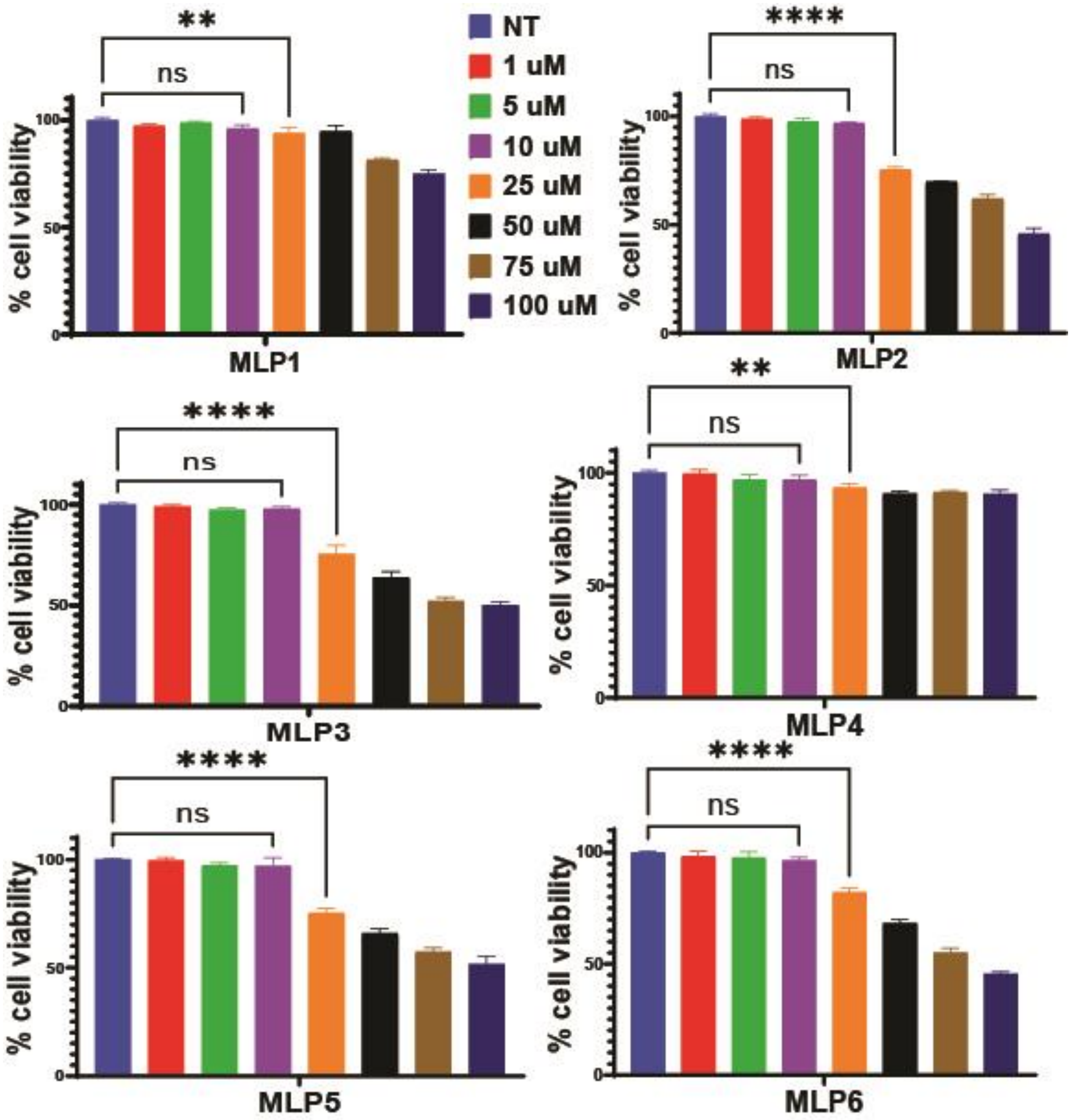

Supplementary Figure S3. Cytotoxicity of peptides in MDA-MB-231 cells at different concentrations (incubation time: 48 h; Graphs represent mean normalized values (based on No Treatment or NT group; n = 3). Error bars indicate standard deviation. ns means non-significant (p-value >0.05, ** means p-value < 0.001, and **** means p-value < 0.0001

Supplementary Figure S4. The complete (unedited) Western blot images provided in the manuscript.

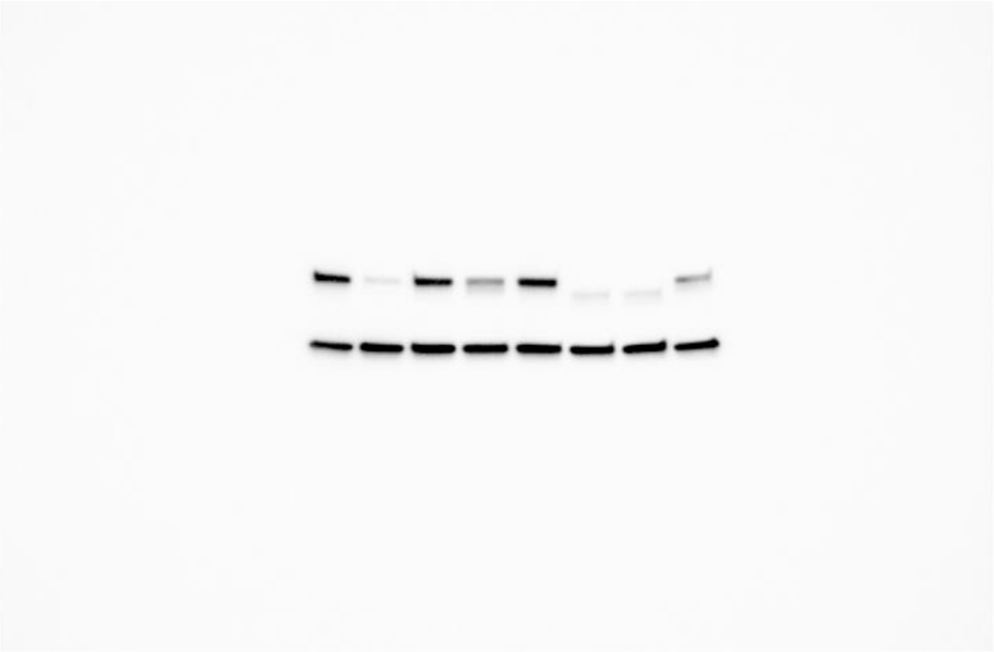

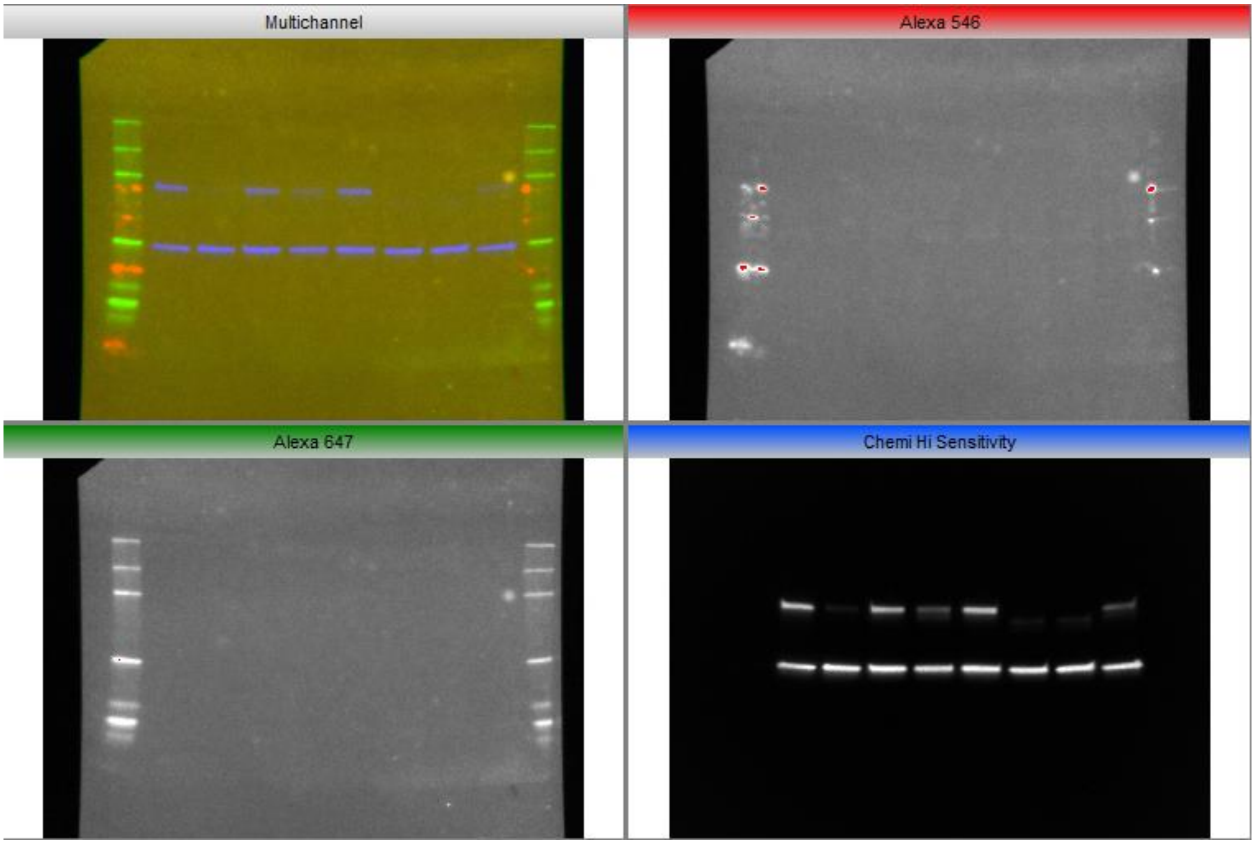

A) Figure 10A

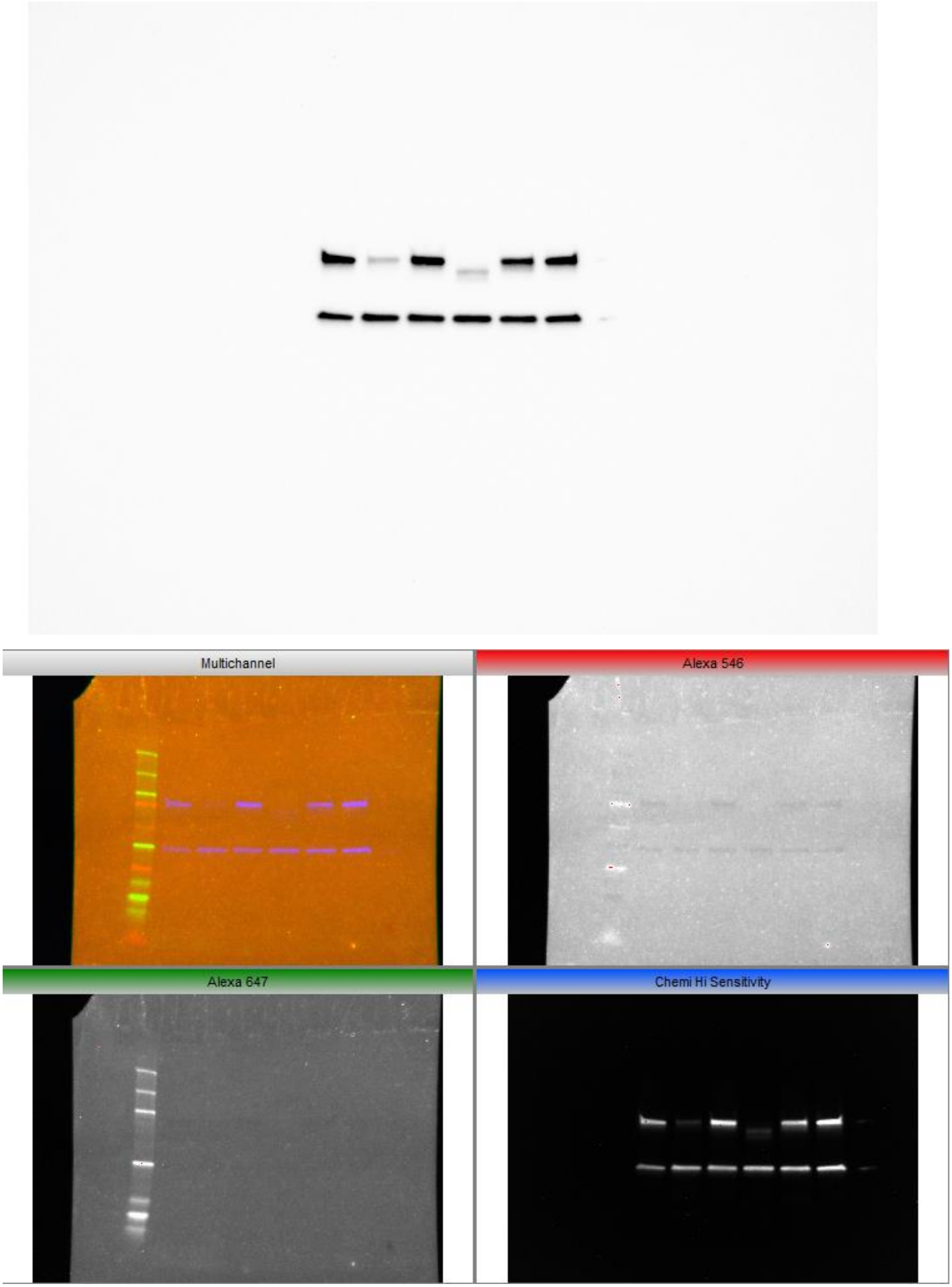

B) Figure 10B

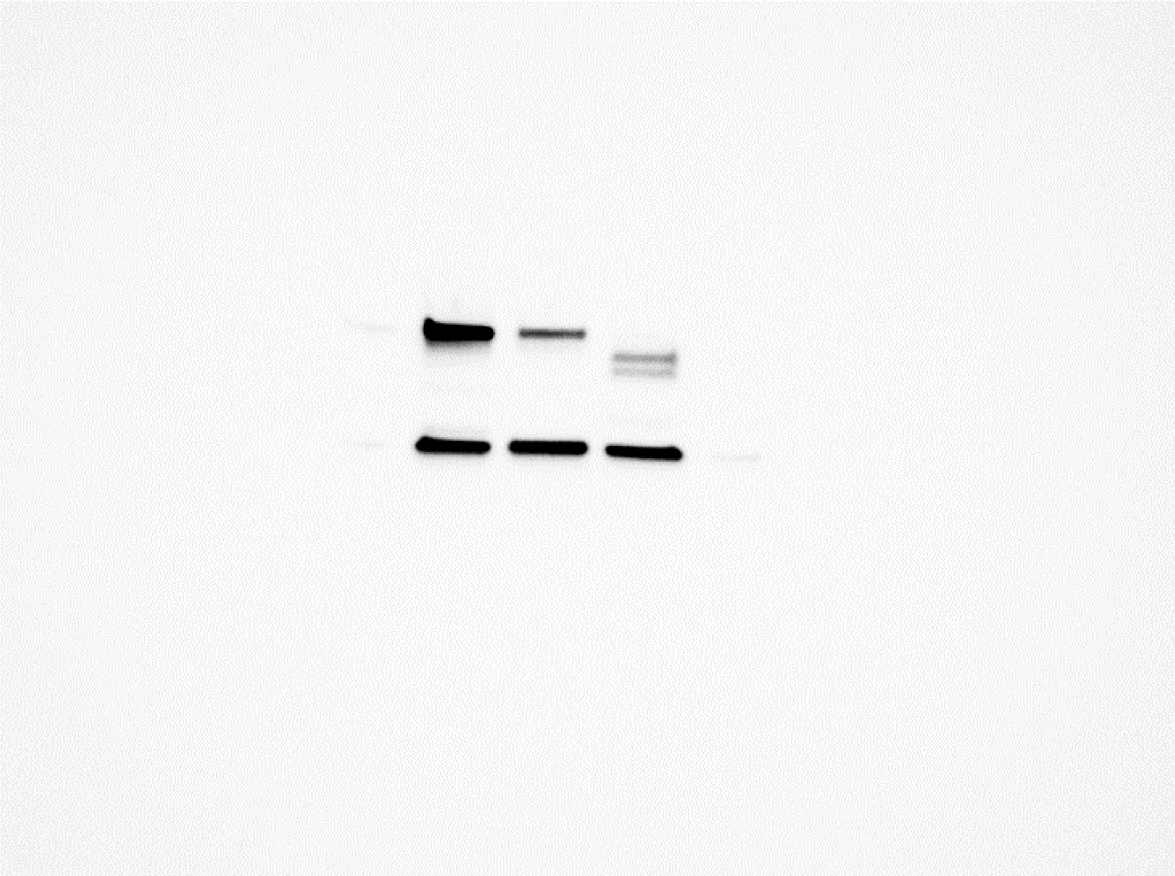

C) Figure 10C
